# Functional classification of *DDOST* variants of uncertain clinical significance in congenital disorders of glycosylation

**DOI:** 10.1038/s41598-023-42178-y

**Published:** 2023-10-17

**Authors:** Sjors M. Kas, Piyushkumar A. Mundra, Duncan L. Smith, Richard Marais

**Affiliations:** 1 Molecular Oncology Group, Cancer Research UK Manchester Institute, The University of Manchester, Wilmslow Road, Manchester, M20 4BX UK; 2 Biological Mass Spectrometry Unit, Cancer Research UK Manchester Institute, The University of Manchester, Wilmslow Road, Manchester, M20 4BX UK; 3 Present Address: Oncodrug Ltd, Alderley Park, Macclesfield, Cheshire, SK10 4TG UK

**Keywords:** Genetics, Molecular biology

## Abstract

Congenital disorders of glycosylation (CDG) are rare genetic disorders with a spectrum of clinical manifestations caused by abnormal *N*-glycosylation of secreted and cell surface proteins. Over 130 genes are implicated and next generation sequencing further identifies potential disease drivers in affected individuals. However, functional testing of these variants is challenging, making it difficult to distinguish pathogenic from non-pathogenic events. Using proximity labelling, we identified OST48 as a protein that transiently interacts with lysyl oxidase (LOX), a secreted enzyme that cross-links the fibrous extracellular matrix. OST48 is a non-catalytic component of the oligosaccharyltransferase (OST) complex, which transfers glycans to substrate proteins. OST48 is encoded by *DDOST*, and 43 variants of *DDOST* are described in CDG patients, of which 34 are classified as variants of uncertain clinical significance (VUS). We developed an assay based on LOX *N*-glycosylation that confirmed two previously characterised *DDOST* variants as pathogenic. Notably, 39 of the 41 remaining variants did not have impaired activity, but we demonstrated that p.S243F and p.E286del were functionally impaired, consistent with a role in driving CDG in those patients. Thus, we describe a rapid assay for functional testing of clinically relevant CDG variants to complement genome sequencing and support clinical diagnosis of affected individuals.

## Introduction

Glycosylation of secreted and membrane-bound proteins is a tightly regulated process that is critical for survival, so complete loss of *N*-linked glycosylation is embryonically lethal in mice^1–3^. In humans, defects in glycosylation are linked to a wide range of diseases including cardiovascular, autoimmune and chronic inflammatory diseases, and to cancer and congenital disorders of glycosylation (CDG)^4–7^. CDG is a collection of rare genetic disorders with an extremely broad spectrum of clinical manifestations and potential driver mutations in more than 130 genes^8^. Abnormal *N*-linked glycosylation causes the majority of CDGs and the disease is divided into two main subtypes: CDG-I where the assembly of lipid-linked oligosaccharides or their transfer to newly synthesised proteins is altered; and CDG-II which is characterised by defects in the processing of the protein-attached oligosaccharides^9^. CDG patients are diagnosed as having a significant neurological component, often presented as developmental delay, and abnormal glycosylation of serum transferrin^10,11^. Next-generation sequencing is an important tool for identifying potential CDG variants at the genomic level^12,13^, but discriminating pathogenic from non-pathogenic variants is challenging and requires systematic testing in relevant models.

*N*-linked glycosylation is initiated by the oligosaccharyltransferase (OST) complex, of which there are two subtypes centered on the catalytic subunits STT3A (STT3A-OST) and STT3B (STT3B-OST)^14–16^. The sub-complexes share 6 non-catalytic subunits, OST4, RPN1, RPN2, TMEM258, DAD1 and OST48 (which is encoded by *DDOST*), but also have unique non-catalytic subunits; OSTC and KRTCAP2 (KCP2) for STT3A-OST and TUSC3 and MAGT1 for STT3B-OST. The OST complexes attach glycans to Asn-Xaa-Ser/Thr (Xaa: any amino acid except proline) motifs on proteins in the endoplasmic reticulum (ER) both co- and post-translationally^4^, and the growing saccharide trees mature further in the ER and Golgi apparatus^4^. *N*-glycosylation affects the structure and function of many cell surface and secreted proteins by modulating processes such as folding, solubility, stability, degradation and activity^17^. Lysyl oxidase (LOX) is a secreted amine oxidase that stabilises the extracellular matrix (ECM) by crosslinking fibrillar elastin and collagens^18,19^. The ECM provides a key structural network that supports many essential cellular processes^20,21^, so defects in the formation and dynamic remodelling of the ECM drives diseases such as connective tissue disorders and cardiovascular diseases, but also fibrosis and cancer^22–24^. The catalytic domain of LOX resides at the C-terminus of the protein and is released from the regulatory N-terminal domain in the ECM by the proteinases BMP-1/procollagen C^19,25,26^. Critically, the N-terminal domain has three *N*-linked glycosylation sites that are required for optimal enzyme activity^25^.

Here, we performed an APEX2-based proximity labelling screen to identify proteins that transiently interact with LOX to improve understanding of ECM biology. From this screen, we identified OST48 as a LOX-interacting protein, and show that CRISPR/Cas9-mediated *DDOST* depletion caused rapid loss of LOX *N*-glycosylation. With this insight, we developed a LOX *N*-glycosylation-based complementation assay to assess functionally the pathogenicity of *DDOST* variants of uncertain clinical significance (VUS) in CDG patients. To validate our assay, we confirmed that the previously characterised variants p.L364Ffs*11 and p.I405Tfs*7 had impaired function. Surprisingly, 39 of the remaining 41 *DDOST* variants had normal function in our assay, but we show that the p.S243F and p.E286del variants, identified by next generation sequencing but untested, had impaired function and so are likely pathogenic. Thus, we describe an assay for rapid functional testing of *DDOST* VUS in CDG to complement genome sequencing and support clinical diagnosis of CDG patients, and our assay could be rapidly adapted to test other components of the OST complex.

## Results

### APEX2-based proximity labelling to identify novel LOX-interactors

**Figure 1:**
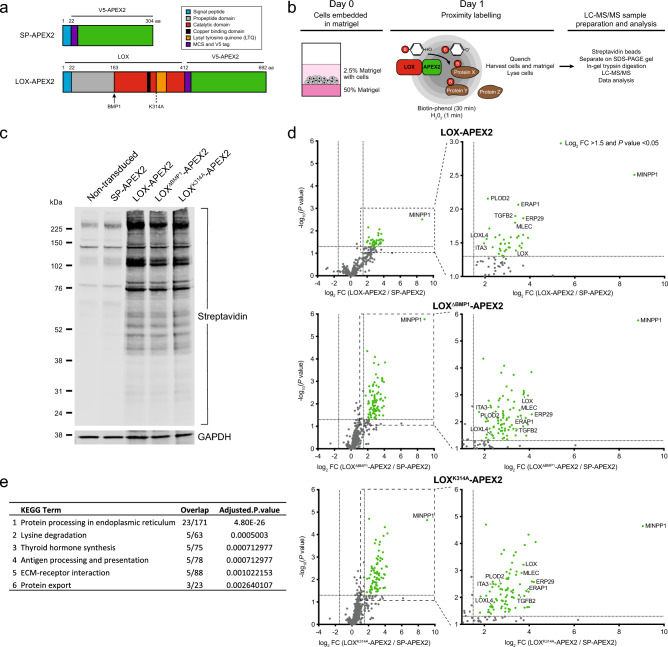
APEX2-based proximity labelling identifies LOX interacting proteins. (**a**) Schematic diagram of SP-APEX2 and LOX-APEX2 constructs, with domain structures, and positions of the BMP1 cleavage site and K314A mutation. Numbers above: amino acids. (**b**) Schematic diagram of APEX2-labelling experimental approach for LOX interacting proteins. (**c**) Western blot for biotinylated proteins revealed by streptavidin binding to immobilised proteins in lysates from MDA-MB-231 cells stably expressing SP-APEX2 or the indicated LOX-APEX2 variants. GAPDH: loading control. Representative image of 3 independent experiments. (**d**) Volcano plots showing proteins interacting with the indicated LOX-APEX2 variants compared to SP-APEX2 controls in MDA-MB-231 cells. Expanded regions highlight the most significant interactors. Green dots: log_2_ abundance fold change (FC) > 1.5, *P* < 0.05). Data are from three independent biological replicates. (**e**) KEGG pathway analysis for the top five significantly enriched pathways for LOX interacting proteins.

To map the LOX interactome and, in particular, identify transient interactions between LOX and other cellular proteins we used APEX2-based proximity labelling^27,28^. We fused the 27 kDa catalytic domain of APEX2 to the C-terminus of full-length wild-type LOX (LOX-APEX2), to LOX lacking the BMP-1 cleavage site (LOX^ΔBMP1^-APEX2), to catalytically inactive LOX (LOX^K314A^-APEX2), and, as a control for APEX2 secretion, to the LOX signal peptide (SP-APEX2) (Fig. 1a).

The LOX-APEX2 variants were stably expressed in human triple negative breast cancer MDA-MB-231 cells and we confirmed that they were secreted as expected, and that LOX^ΔBMP1^-APEX2 was not cleaved to the mature active form (Supplementary Fig. 1a,b). To provide an appropriate cellular context, we embedded the cells in Matrigel for 24 h, incubated with biotin-phenol (BP) for 30 min, and then with hydrogen peroxide (H_2_O_2_) for 1 min (Fig. 1b). To release both intracellular and extracellular biotinylated proteins, the matrigel and cells were harvested and lysed together. Protein blotting with streptavidin confirmed enrichment of biotinylated proteins in cells expressing the LOX-APEX2 variants compared to SP-APEX2 (Fig. 1c).

Next, the biotinylated proteins were captured on streptavidin beads and identified by liquid chromatography-tandem mass spectrometry (LC–MS/MS). Compared to cells expressing the SP-APEX2 control, we identified 37 proteins enriched in LOX-APEX2 cells, 85 in LOX^ΔBMP1^-APEX2 cells and 88 in LOX^K314A^-APEX2 cells (Fig. 1d, Supplementary Fig. 1c and Supplementary Table 1). Importantly, these included LOX itself, transforming growth factor beta-2 (TGFB2), integrin alpha-3 (ITGA3) and lysyl oxidase-like protein 4 (LOXL4), known to be involved in LOX biology, which provided assurance in our experimental approach^19,29,30^. We also identified the ECM proteins AGRN, COL6A1, ITGA2 and LAMB3, which also provide confidence in our experimental approach (Supplementary Fig. 1d). Intriguingly, KEGG pathway enrichment analysis revealed that the most enriched group of proteins (23 proteins) were implicated in protein processing in the endoplasmic reticulum (Fig. 1e, Supplementary Fig. 1e).

### *DDOST* regulates LOX expression

Thus, in addition to ECM proteins, we identified components of ER quality control processes as LOX-interactors (Supplementary Fig. 1c,d), so we used CRISPR/Cas9-based screening to examine how these proteins regulated LOX expression. First, we validated our antibody by showing that two selective sgRNAs depleted LOX by > 95% in MDA-MB-231 cells (Supplementary Fig. 2a). Note that we observed two non-specific bands that were not lost when *LOX* was depleted, and which were apparent in some, but not all western blots. Next, we established our assay conditions and show that, in agreement with previous studies^31–33^, LOX was strongly induced by hypoxia (1% O_2_ for 24 h) in MDA-MB-231 cells (Supplementary Fig. 2b). Notably, oxygen consumption increases at higher cell densities^34^, and accordingly we show that LOX expression increased when MDA-MB-231 cells were grown at higher cell density (Supplementary Fig. 2b). We also confirmed hypoxia induced LOX in U87 human glioblastoma cells, as reported previously^35^, and we show that LOX is hypoxia-induced in human pancreatic fibroblasts (Supplementary Fig. 2c).

**Figure 2:**
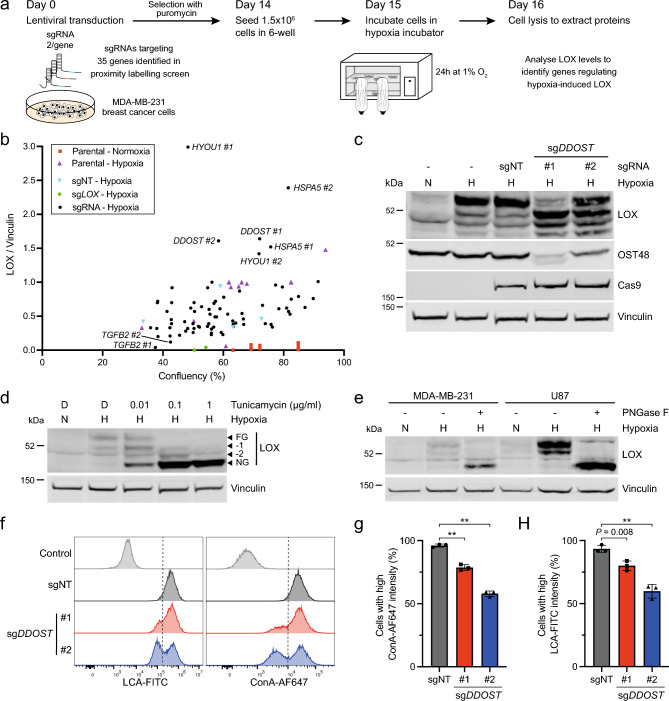
*DDOST* depletion suppresses LOX and cell surface *N*-glycosylation. (**a**) Schematic diagram of genome editing experimental approach to test how depletion of LOX-interactors affect LOX expression in hypoxic MDA-MB-231 cells. Cells were transduced with LentiCRISPRv2-sgRNA vectors targeting 35 genes identified in Fig. 1d (LOX-APEX2 interactors), re-seeded 14 days later, transferred to 1% O_2_ the next day and harvested for western blot analysis 24 h later. (**b**) Graph showing LOX expression (fold change compared to vinculin) as a function of cell confluency (%) in MDA-MB-231 cells after CRISPR/Cas9 gene editing for 35 genes selected from Fig. 1d (LOX-APEX2 interactors). Two optimal sgRNAs per gene were used alongside three independent non-targeting controls (sgNT; cyan) and two sg*LOX* (green) controls. Cell confluency was determined by IncuCyte imaging 15 days after transduction, then the cells were placed in 1% O_2_ for 24 h (except the MDA-MB-231 parental control; red) and harvested for LOX quantification by western blot. (**c**) Immunoblot for LOX, OST48, Cas9 and vinculin (loading control) in MDA-MB-231 cells after CRISPR/Cas9 gene editing with sg*DDOST* (#1, #2) or sgNT (control) for 15 days and then incubated in normoxia (N) or hypoxia (H; 1% O_2_) for 24 h. Image representative of 3 independent experiments. (**d**) Western blot for LOX and vinculin (loading control) in MDA-MB-231 cells treated with the indicated concentrations of tunicamycin or DMSO (D) control and incubated in normoxia (N) or hypoxia (H; 1% O_2_) for 24 h. FG: fully *N*-glycosylated; NG: non-glycosylated; −1, −2 incomplete *N*-glycosylation (intermediate). Image representative of 3 independent experiments. (**e**) Western blot for LOX and vinculin (loading control) in PNGase F- (+) or vehicle-treated (-) lysates from MDA-MB-231 and U87 cells incubated in normoxia (N) or hypoxia (H; 1% O_2_) for 24 h. Image representative of 3 independent experiments. (**f**) Histograms showing LCA and ConA binding in U87 cells 14 days after CRISPR/Cas9 gene editing with sg*DDOST* (#1, #2) or sgNT (control). The control shows unstained cells and data are representative of 3 independent experiments. (**g**, **h**) Quantification of ConA (g) and LCA (h) binding in U87 cells 14 days after CRISPR/Cas9 gene editing with sg*DDOST* (#1, #2) or sgNT (control). Data are mean ± s.d. of 3 independent experiments. ** *P* < 0.001.

For the screen, we selected 37 of our LOX-interactors (LOX-APEX2; Supplementary Fig. 1c), of which 35 had CRISPR/Cas9-targetable genes. We selected 2 optimal sgRNAs per gene from the MinLibCas9 Library and 3 non-targeting sgRNA controls^36^. The sgRNAs were introduced independently into MDA-MB-231 cells using lentiviral transduction, and 14 days later 1.5 × 10^6^ cells were seeded into 6-well plates and transferred to hypoxia the next day for 24 h after which LOX was quantified by immunoblotting (Fig. 2a). We confirmed that higher confluency caused increased LOX expression and used the *LOX* sgRNAs to provide a control for LOX depletion (Fig. 2b). To validate our approach, in accordance with previous studies^29,30^, we confirmed that *TGFB2* depletion suppressed LOX expression (Fig. 2b). We show that depletion of the heat shock protein 70 (HSP70) family members *HYOU1* and *HSPA5* (also known as *BiP*) increased LOX expression. *DDOST* depletion also increased LOX expression to variable amounts, but a more intriguing and more robust observation was that it caused LOX to migrate as a series of smaller bands with apparent masses of ~ 48,000, ~ 50,000 and ~ 52,000 dalton (Da) in SDS-PAGE (Fig. 2c).

### OST48 is required for LOX and cell surface *N*-glycosylation

We were intrigued by the impact of *DDOST* gene editing on LOX migration in SDS-PAGE, because *DDOST* encodes OST48, a non-catalytic 48 kDa subunit of the OST complex. We hypothesised that OST48 depletion impaired LOX *N*-glycosylation. To test this first we show that tunicamycin, an inhibitor of the first step in *N*-linked oligosaccharide biosynthesis, reduced LOX from an apparent mass of ~ 54,000 to bands of ~ 48,000, ~ 50,000 and ~ 52,000 in SDS-PAGE in both MDA-MB-231 and U87 cells (Fig. 2d, Supplementary Fig. 2d). Furthermore, when MDA-MB-231 and U87 cell lysates were treated with PNGase F, an enzyme that cleaves *N*-linked oligosaccharides from glycoproteins, LOX migrated at ~ 48,000 (Fig. 2e). Thus, fully glycosylated (FG) LOX had a mass of ~ 54,000, and non-glycosylated (NG) LOX a mass of ~ 48,000 and, consistent with previous studies reporting the presence of three *N*-linked glycosylation sites in LOX^25,37^, we observed intermediate glycosylation forms of ~ 52,000 (−1) and 50,000 (−2). Our data are consistent with the reduced LOX migration in SDS-PAGE when *DDOST* was depleted being due to full or partial loss of LOX *N*-glycosylation.

To test if *DDOST* depletion caused broader reduction in cellular *N*-glycosylation, we transduced MDA-MB-231 and U87 cells with sgRNAs targeting two different genomic regions of *DDOST* (sg*DDOST*) and a non-targeting control (sgNT). After 14 days, we analysed cell surface glycans using the fluorescently labelled lectins Concanavalin A (ConA) to detect α-linked mannose and *Lens Culinaris* agglutinin (LCA) to detect α-linked mannose and core fucosylated *N*-glycans. Both U87 and MDA-MB-231 cells transduced with sg*DDOST* showed decreased ConA and LCA binding compared to cells transduced with the sgNT control (Fig. 2f–h, Supplementary Fig. 2e–g).

### A functional OST complex is required for cell proliferation

**Figure 3:**
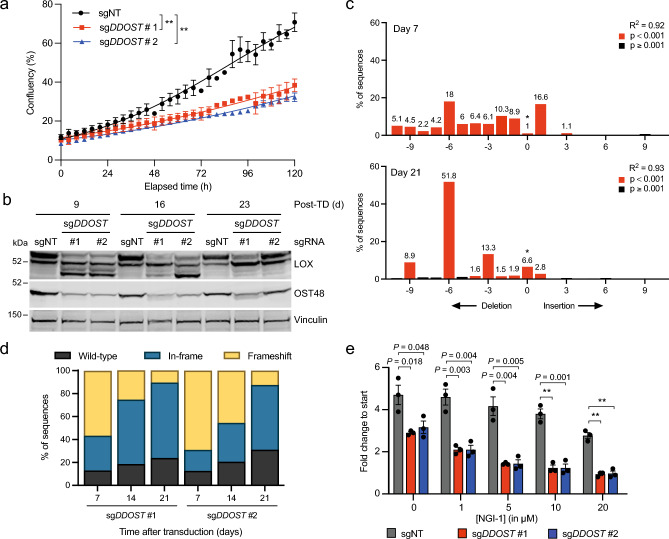
Functional OST48 is essential for cell survival. (**a**) Proliferation of U87 cells after CRISPR/Cas9 gene editing with sg*DDOST* (#1, #2) or sgNT (control). Cells were re-seeded 7 days after transduction and proliferation was determined from day 8 for 120 h using real-time IncuCyte imaging. Data are mean ± SEM of three independent experiments with three technical repeats per condition. ** *P* < 0.001; F test for comparison of logistic growth curve fits. (**b**) Western blot for LOX, OST48 and vinculin (loading control) in U87 cells after CRISPR/Cas9-mediated gene editing with sg*DDOST* (#1, #2) or sgNT 9, 16 and 23 days after transduction. Cells were re-seeded 7, 14 and 21 days after transduction and transferred to hypoxia (1% O_2_) the next day for 24 h. Data are representative of 3 independent experiments. (**c**) TIDE analyses of the region of *DDOST* targeted by sg*DDOST* (#1 used) 7 or 21 days after transduction. The graph shows the fraction of unmodified (*; wild-type) and modified sequences (red: *P* < 0.001; black: *P* ≧ 0.001). Data are representative of 3 independent experiments. (**d**) Quantification of TIDE analysis of the region of *DDOST* targeted by sg*DDOST* (#1, #2) 7, 14 and 21 days after transduction compared to sgNT control. Data are mean ± s.d. of three independent experiments. In-frame indels (including unmodified alleles) on day 21 compared to day 7: *P* = 0.027 for sg*DDOST* #1; *P* = 0.008 for sg*DDOST* #2 (paired two-tailed Student’s *t* tests). (**e**) Graph showing U87 cell proliferation after CRISPR/Cas9 gene editing with sg*DDOST* (#1, #2) or sgNT (control) followed by treatment with NGI-1 at the indicated concentrations. Cells were seeded 7 days after sgRNA transduction, NGI-1 was added on day 8 and proliferation was measured for 4 days using real-time IncuCyte imaging. Data are mean ± SEM of three independent biological replicates with three technical repeats per condition. ** *P* < 0.001.

Our data above show that OST48 is essential for LOX *N*-glycosylation, and notably we show that *DDOST* depletion suppressed U87 and MDA-MB-231 cell proliferation (Fig. 3a, Supplementary Fig. 3a), confirming previous data that OST48 is essential for eukaryotic cell viability^38^. Interestingly, the residual OST48 expression seen at 7 and 14 days was almost completely restored to sgNT levels 21 days after gene targeting (Supplementary Fig. 3b), and this coincided with a recovery in LOX *N*-glycosylation (Fig. 3b). We used tracking of insertions and deletions (indels) by decomposition (TIDE) to quantify genetic events at the targeted site of *DDOST* to confirm that our CRISPR/Cas9 targeting of *DDOST* was highly efficient in U87 cells 7 days after gene editing (Fig. 3c,d)^39^. Interestingly however, whereas in-frame indels (including unmodified alleles) accounted for only 30.9% of genome sequences on day 7, that increased to 87.5% by day 21 (sg*DDOST* #2, *P* = 0.008; paired two-tailed Student’s *t* test) (Fig. 3c,d). Thus, cells expressing wild-type OST48 have a proliferative advantage over *DDOST*-depleted cells, confirming the essentiality of OST48^38,40^. Finally, we treated U87 cells with NGI-1, an inhibitor of STT3A and STT3B. NGI-1 reduced U87 cell proliferation in a dose dependent manner, and this response was more pronounced when *DDOST* was depleted (Fig. 3e). Collectively, our data show that OST48 is an indispensable component of the OST complex and that its depletion increases sensitivity to catalytic subunit inhibition.

### Pathogenic *DDOST* variants affect LOX *N*-glycosylation

**Figure 4:**
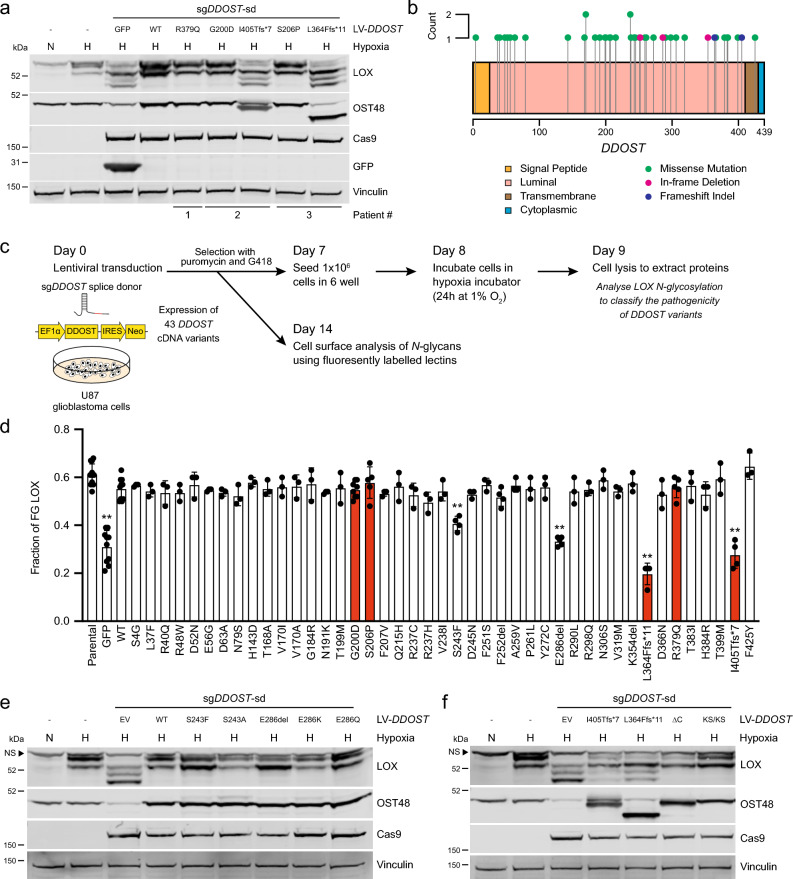
LOX *N*-glycosylation assay enables functional testing of *DDOST* variants. (**a**) Western blot for LOX, OST48, Cas9, GFP and vinculin (loading control) in U87 cells after CRISPR/Cas9 gene editing with a sgRNA targeting the *DDOST* splice donor site (sg*DDOST*-sd) and reconstituted with the indicated *DDOST* cDNA variants. Cells were re-seeded 7 days after transduction, then moved to normoxia (N) or hypoxia (H; 1% O_2_) on day 8 for 24 h. Data are representative of 3 independent experiments. (**b**) Lollipop plot of 43 *DDOST* variants collected from publicly available reports and the ClinVar database. (**c**) Schematic diagram of the experimental approach for the cDNA-based complementation assay to determine the impact of *DDOST* variants on LOX *N*-glycosylation and cell surface *N*-glycan levels in U87 cells. (**d**) Quantification of fully *N*-glycosylated (FG) LOX as a fraction of total LOX expression in U87 cells after CRISPR/Cas9 gene editing with sg*DDOST*-sd and reconstituted with GFP, *DDOST* (WT) and the 43 *DDOST* cDNA variants indicated. Parental cells are shown for reference. Cells were re-seeded 7 days after transduction and moved to hypoxia (1% O_2_) on day 8 for 24 h. Variants in red were reported previously^13,41,42^. Data are plotted mean ± s.d. of at least three independent experiments. ** adjusted *P* values < 0.001 for variants compared to WT; unpaired two-tailed Student’s *t* tests with FDR multiple testing correction. (**e**, **f**) Western blots for LOX, OST48 and vinculin (loading control) in U87 cells after CRISPR/Cas9 gene editing with sg*DDOST*-sd and reconstitution with luminal (**e**) or C-terminal (**f**) *DDOST* variants. Cells were re-seeded 7 days after transduction, then moved to normoxia (N) or hypoxia (H; 1% O_2_) on day 8 for 24 h. Images are representative of 3 independent experiments each. Arrowheads highlight non-specific (NS) bands.

Impaired glycosylation is a hallmark of CDG, but the identification of the disease-causing mutations is challenging. Since OST48 is essential for LOX *N*-glycosylation, we tested if we could use this in a functional complementation assay to discriminate benign from pathogenic *DDOST* VUS. As proof of principle, we complemented *DDOST*-depleted U87 cells with wild-type *DDOST* cDNA and five mutations reported in 3 DDOST-CDG patients^13,41,42^, and analysed the impact on LOX *N*-glycosylation under hypoxic conditions. We used CRISPR/Cas9 to target the *DDOST* splice donor (sg*DDOST*-sd) site at exon 2 to disrupt endogenous *DDOST* and in parallel complemented the cells with a *DDOST* cDNA that is intrinsically resistant to this sgRNA. We validated this approach by confirming that in U87 cells targeted with sg*DDOST*-sd, LOX *N*-glycosylation was not restored by GFP but was restored by wild-type *DDOST* (Fig. 4a). LOX *N*-glycosylation was also restored by *DDOST* p.G200D, p.S206P and p.R379Q, but not by p.L364Ffs*11 or p.I405Tfs*7, mutations that truncate the C-terminus of OST48 (Fig. 4a). Thus, in our assay two patients each had one mutation that impaired OST48 function, but the homozygous mutation of the other patient appeared to be fully active. Notably, in mice heterozygous *Ddost* knockout is tolerated whereas homozygous loss is embryonic lethal^43^. Collectively, our data support a hypothesis that residual OST48 function from a normal or hypomorphic *DDOST* allele is essential for survival and through analysis of LOX *N*-glycosylation, we can rapidly test the function of *DDOST* variants from CDG patients.

To validate the potency and scalability of this assay, we collected all publicly available *DDOST* variants from CDG case reports and the ClinVar database^44^, which aggregates human genetic CDG variants and their interpretations of their significance to disease. We identified 43 *DDOST* variants, which are spread throughout the gene and include 38 missense mutations, 1 frameshift deletion, 1 frameshift insertion, and 3 in-frame deletions (Fig. 4b, Supplementary Table 2). Notably, the only 2 variants that are annotated in ClinVar as having clinical significance were identified in the first reported DDOST-CDG patient^13^, and the functional validation of the impaired *N*-glycosylation caused by the mutations required the derivation of a fibroblast cell line from the patient. Of the remaining 41 variants, 34 are classified as VUS (Supplementary Table 2).

To test the clinical significance of these VUS, we used site-directed mutagenesis to generate cDNA expression constructs for the 43 *DDOST* variants. U87 cells were simultaneously transduced with sg*DDOST*-sd and the *DDOST* cDNA constructs followed by concurrent puromycin and neomycin selection (Fig. 4c). The cells were incubated for 7 days to ensure optimal depletion of endogenous *DDOST* and to limit the number of cells re-expressing wild-type OST48, and then re-seeded, subjected to hypoxia for 24 h, and the fraction of FG LOX in the cells was quantified (Fig. 4c). We validated that the 5 variants yielded similar results to previously tested (Fig. 4a), with *DDOST* p.G200D, p.S206P and p.R379Q restoring LOX *N*-glycosylation, but p.L364Ffs*11 and p.I405Tfs*7 failing to do so (Fig. 4d). Thirty-six of the other variants restored LOX *N*-glycosylation, but p.S243F and p.E286del consistently showed a partial loss (intermediate effect) of LOX *N*-glycosylation (Supplementary Fig. 4a).

Next, we used our approach to examine the impact of the *DDOST* VUS on general cell surface *N*-glycans by staining for ConA and LCA binding (Fig. 4c). In contrast to our LOX *N*-glycosylation assay, we did not observe any impact of *DDOST* depletion on ConA or LCA binding after 7 days, saw a 26–44% reduction at 14 days, but this was fully restored at 21 days (Supplementary Fig. 4b). We therefore screened the 43 variants after 14 days, and in this assay only p.L364Ffs*11 and p.I405Tfs*7 reduced ConA and LCA binding, whereas all other variants did not, including p.S243F and p.E286del (Supplementary Fig. 4c,d). Thus, *DDOST* depletion had a much more subtle effect on general cell surface protein *N*-glycosylation than on LOX *N*-glycosylation.

### The C-terminal domain is critical for OST48 function

Finally, we sought to understand how the *DDOST* variants in CDG affected OST48 biological activity. OST48 is a transmembrane protein with the majority of the protein in the lumen of the ER (Fig. 4b)^45–47^, and pathogenic variants p.S243F and p.E286del are in the luminal region. S243 is predicted to form 5 hydrogen bonds with proximal residues, 2 of which would be abrogated by the S243F change (Supplementary Fig. 4e). Moreover, the bulky hydrophobic side group of phenylalanine in place of the small side group of serine could cause steric hindrance. To test which effect disrupts OST function, we mutated S243 to alanine (p.S243A) to remove the hydrogen bonds to proximal residues made by the serine hydroxyl group. *DDOST* p.S243A still restored LOX *N*-glycosylation (Fig. 4e, Supplementary Fig. 4f), suggesting that it is the bulky side group of phenylalanine rather than the loss of the hydrogen bonds that impairs OST48 function in this variant. E286 is predicted to link the N-terminal domain (NTD) to a β-sheet of the central domain that is suggested to be important for the interaction of OST48 with STT3A/B and RPN2^15,48^. We therefore tested if deletion of E286 or loss of the charge was responsible for the pathogenicity of the p.E286del variant (Supplementary Fig. 4g). We mutated E286 to lysine (p.E286K) and glutamine (p.E286Q) to replace the acidic residue with a basic or uncharged side chain, respectively. Notably, both restored LOX *N*-glycosylation (Fig. 4e, Supplementary Fig. 4f), showing that it is loss of the amino acid at position 286 rather than loss of the interactions driven by the acidic residue that is critical for the pathogenicity of this variant.

The C-terminus of OST48 includes a transmembrane anchor and a 9 amino acid cytosolic tail containing an ER retention signal centered around K435 and K437 and a DAD1 interaction site (Fig. 4b)^45–47^. Both the transmembrane region and cytosolic tail are lost with the pathogenic variants p.L364Ffs*11 and p.I405Tfs*7 (Fig. 4a), so to determine which C-terminal domain of OST48 is critical for function, we mutated K435 and K437 to serines (KS/KS) and generated a second mutant lacking the 9 amino acid cytosolic tail (∆C). Notably, both mutations had an intermediate impact on LOX *N*-glycosylation (Fig. 4f, Supplementary Fig. 4f), indicating that both the transmembrane region and the cytosolic tail are important for OST48 activity. Consistent with the critical role of the cytosolic tail, we note that addition of a V5 tag to the C-terminus of OST48 also impaired LOX *N*-glycosylation in our assay (Supplementary Fig. 4a).

### A functional OST complex is critical for LOX *N*-glycosylation

**Figure 5:**
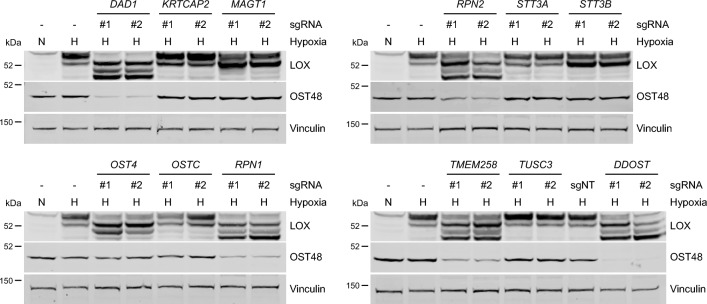
Independent depletion of each common OST subunit suppresses LOX *N*-glycosylation and OST48 expression. Western blots for LOX, OST48 and vinculin (loading control) 12 days after CRISPR/Cas9-mediated genome targeting for the indicated OST genes in U87 cells. Samples were re-seeded 10 days after transduction and moved to normoxia (N) or hypoxia (H; 1% O_2_) on day 11 for 24 h.

Thus, our functional complementation assay allows rapid and robust testing and exploration of the functional impact of *DDOST* VUS in CDG patients. Notably, several other OST genes are also mutated in CDG patients (Supplementary Table 3), so we explored if our assay could be used to test these VUS. Interestingly, sgRNAs that targeted the common subunits, *OST4*, *RPN1*, *RPN2*, *TMEM258* and *DAD1* all affected LOX *N*-glycosylation to a similar extent to *DDOST* depletion in U87 and MDA-MB-231 cells (Fig. 5, Supplementary Fig. 5a). In contrast, sgRNAs targeting *STT3B* and *MAGT1* had an intermediate effect on LOX *N*-glycosylation, and sgRNAs that targeted *STT3A*, *OSTC*, *KRTCAP2* and *TUSC3* had no impact (Fig. 5, Supplementary Fig. 5a). OST48 and DAD1 are reported to be essential for the stability of the entire OST complex^49^. We confirmed that *DDOST* depletion resulted in loss of RPN1 and RPN2 (Supplementary Fig. 5b,c). We extended this observation by showing that sgRNAs targeting *TMEM258*, *DAD1*, *RPN1*, *RPN2* and *OST4* all caused loss of OST48, whereas sgRNAs targeting *STT3A*, *STT3B*, *OSTC*, *KRTCAP2*, *TUSC3* or *MAGT1* did not affect OST48 expression (Fig. 5, Supplementary Fig. 5a). Thus, we conclude that our assay could be extended to test VUS in most, but not all components of the OST complex.

## Discussion

We use APEX2-based proximity labelling to identify LOX-interactor proteins. In addition to proteins previously linked to LOX biology, we also identified extracellular proteins and components of the quality control pathways for newly synthesised proteins in the ER. Of note, we identified OST48, a non-catalytic subunit of the OST complex, as a LOX-interactor, and we used CRISPR/Cas9 gene editing to confirm that OST48 is critical for LOX *N*-glycosylation. Curiously, we did not identify other proteins from the OST complex as LOX-interactors, but using CRISPR/Cas9, we established that the common OST subunits TMEM258, DAD1, RPN1, RPN2 and OST4 were essential for LOX *N*-glycosylation, whereas the catalytic subunits STT3A and STT3B, and the unique subunits OSTC, KRTCAP2, TUSC3 and MAGT1 were not. In part this suggests that depletion of any common subunit causes instability of both subcomplexes directly or indirectly via OST48 loss, whereas depletion of the catalytic and unique subunits did not. Note however, that depletion of *STT3B* and *MAGT1* did have a minor impact on LOX *N*-glycosylation, suggesting that this is the preferred subcomplex that drives LOX *N*-glycosylation, but that STT3A-OST can also partially perform this task. These data suggest that in addition to recruiting lipid-linked oligosaccharide (LLO) molecules and stabilising the OST complex^15,48–52^, OST48 is proximal to the newly synthesised LOX for *N*-glycosylation to occur. However, the mechanism whereby OST48 facilitates the interaction between the recipient polypeptide and the OST complex is unclear.

Large-scale genomic sequencing has revealed genomic variants that drive human diseases, including for rare genetic diseases such as CDG^53^. However, it is still challenging to discriminate disease-causing from non-disease variants, so the majority of new variants are designated VUS. Analysis of large cohorts of affected individuals may reveal genotype–phenotype correlations, but because patient numbers are small and there is enormous genetic heterogeneity, correlations such as these are difficult to establish for CDG. Hypoglycosylation has been confirmed in patient-derived fibroblasts to validate a small number of *DDOST* variants as pathogenic^13,42^, but complementation with wild-type *DDOST* cDNA in patient-derived fibroblasts is laborious and challenging and does not lend itself to a robust and effective basis for an assay^13^. Moreover, this approach does not allow testing of individual VUS in patients with different mutations on the two *DDOST* alleles. Thus, an assay based on a specific biomarker that allows side by side characterisation of the pathogenicity of each VUS independently would be an important advance in CDG diagnosis.

The assay we describe allows rapid and robust testing of *DDOST* VUS based on LOX* N*-glycosylation in only 4–6 weeks. Critically, because LOX is secreted, it is constantly passing through the ER and Golgi, so the population of LOX proteins being measured are transient and constantly changing, providing a dynamic measure of OST complex activity that lasts for several weeks. In contrast, the levels of general cell surface *N*-glycans were more stable, possibly because cells stop proliferating when OST48 is lost, so the turnover of these proteins is reduced. This makes it difficult to assess the impact of OST48 function, and therefore to distinguish pathogenic from non-pathogenic VUS. However, with our cell surface *N*-glycans assay, we tested 43 *DDOST* variants and confirmed that two previously described variants were pathogenic, but another 39 did not have impaired activity in our assay. We did however identify two new VUS that had impaired activity with LOX *N*-glycosylation. This demonstrates that different assays have different sensitivities in defining the activity of specific VUS, and we note that mass spectrometry-based techniques that characterise specific cell surface carbohydrate structures could provide further novel insight into distinct substrate specificities of particular *DDOST* variants^54^. Critically, unlike patient-derived fibroblasts our assay allowed us to test the severity of specific variants from patients carrying two distinct mutations and because our approach tests the VUS in a *DDOST*-null population, artifacts related to compensation from a wild-type allele are minimised. Finally, we show that LOX *N*-glycosylation is also affected by depletion of the common subunits *TMEM258*, *RPN1*, *RPN2*, *OST4* and *DAD1*, suggesting that CDG VUS in these genes could also be tested functionally using our approach.

In addition to exploring the pathogenicity of VUS, we also used our assay to explore the function of specific domains and specific amino acids in OST48. The previously reported pathogenic variants (p.L364Ffs*11 and p.I405Tfs*7) truncate the C-terminal transmembrane region and cytosolic tail of OST48, and we used our assay to show that both the transmembrane region and cytosolic tail are essential for function. The importance of this short tail was emphasised by showing that addition of a V5 epitope tag was sufficient to interfere with OST48 function. In this context, we note that the cytosolic tail interacts with DAD1 and acts as an ER retention signal^45–47^. The two new pathogenic variants we identified were in the ER luminal domain of OST48, so we used our assay to explore how they affected the protein’s function. S243, in the NTD, is predicted to form 5 hydrogen bonds with proximal residues, but we showed that 2 of these are not needed, so it is likely the steric hindrance caused by the large hydrophobic side-group of phenylalanine that abrogates OST48 function. E286 links the α-helix of the NTD to a β-sheet of the central domain and is thought to be important for the interaction with STT3A/B and RPN2^15,48^. Our data shows that it was loss of the amino acid at position 286 rather than loss of the interactions driven by the acidic residue that impaired the function of the protein.

Although our assay can be used to define pathogenicity of VUS, we note that it had limitations because we could not discriminate benign from hypomorphic variants (p.G200D, p.S206P and p.R379Q). First, we used a lentiviral expression vector with the strong EF-1α promoter to express our cDNA constructs to achieve levels of expression close to endogenous OST48, but we cannot exclude that overexpression of hypomorphic variants could mask their pathogenicity. Second, U87 glioblastoma cells may not provide an appropriate physiological background. Third, our system does not allow co-testing of variants, that could affect each other. Fourth, as our approach is cDNA-based, it does not allow testing of variants that affect mRNA splicing, translation or stability. These challenges could be met by combining measurements of LOX *N*-glycosylation with strategies such as CRISPR-Select and saturation genome editing^55,56^, which allow rapid and accurate functional testing of complex genetic sequence variants. Nevertheless, our assay did allow rapid testing of all *DDOST* VUS reported in ClinVar without the need for large libraries of repair templates or expensive deep sequencing data.

In summary, we report a strategy for functional testing of *DDOST* VUS in CDG that is likely suitable for testing variants in other OST genes. It is challenging to integrate functional validation of VUS with existing clinical variant classification guidelines, but our approach provides increased understanding of CDG biology and could be used to support clinical diagnosis in CDG patients.

## Methods

### Plasmids, cloning and site-directed mutagenesis

The V5-APEX2 cassette was PCR amplified with BamHI and NotI sites from pCDNA3_Sec61B-V5-APEX2 (83411, Addgene), a gift from Hyun-Woo Rhee ^57^, and substituted for eGFP in SP-eGFP-N1 and the MsLOX-eGFP-N1 variants as described previously ^58^. LV-EF-1α-IRES-Puro (85132, Addgene) was a gift from Tobias Meyer ^59^. SP-V5-APEX2 and the three LOX-V5-APEX2 variants were subsequently PCR amplified with NotI sites and inserted into LV-EF-1α-IRES-Puro to generate LV-EF-1α-SP-V5-APEX2-IRES-Puro, LV-EF-1α-LOX-V5-APEX2-IRES-Puro, LV-EF-1α-LOX^ΔBMP1^-V5-APEX2-IRES-Puro and LV-EF-1α-LOX^K314A^-V5-APEX2-IRES-Puro. LV-EF-1α-IRES-Neo was generated by replacing the IRES-Puromycin cassette from LV-EF-1α-IRES-Puro (SmaI and HpaI sites) with the IRES-Neomycin resistance cassette (SmaI and EcoRV sites) from Tet-pLKO-Neo (21916, Addgene), a gift from Dmitri Wiederschain ^60^. To generate the *DDOST* (NM_005216.5) cDNA sequence, total RNA was extracted from MDA-MB-231 cells using the RNeasy Plus Mini Kit (74134, Qiagen) and reverse transcribed to cDNA using the QuantiTect Reverse Transcription Kit (205311, Qiagen) with *DDOST* primers according to the manufacturer’s protocol. The NotI sites and the C-terminal V5 epitope tag to generate *DDOST-V5* were added by PCR with flanking primers. GFP was PCR amplified with NotI sites from the MsLOX-eGFP-N1 vector. The GFP and *DDOST* cDNAs were inserted as NotI fragments into the LV-EF-1α-IRES-Neo vector. All PCR reactions were performed using Phusion® High-Fidelity DNA Polymerase (M0530, New England Biolabs) according to the manufacturer’s protocol. LentiCRISPR v2 (52961, Addgene) was a gift from Feng Zhang^61^. All sgRNA sequences were cloned into LentiCRISPR v2 as previously described^61^. Point mutations, deletions and insertions in *DDOST* were introduced using the QuikChange Lightning Site-Directed Mutagenesis Kit (210519, Agilent) according to the manufacturer’s protocol. Site-directed mutagenesis primers were generated using Agilent’s QuikChange Primer Design, of which some were optimised as described previously^62^. All vectors were verified using Sanger sequencing. Primers are listed in Supplementary Table 4. Concentrated lentiviral stocks were produced by transient co-transfection of psPAX2, pMD2.G and the expression vector plasmid in HEK293T packaging cells using the calcium phosphate transfection method. The MD2.G envelope expressing vector and sPAX2 packaging vector were gifts from Didier Trono (plasmids #12259 and 12260, Addgene).

### Cell lines and culture conditions

MDA-MB-231, U87 (U-87MG) and HEK293T (CRL-3216) cells were purchased from American Type Culture Collection (ATCC). Human primary pancreatic fibroblasts (hPaFs) were purchased from Generon (H-6201). All cells were cultured in Dulbecco's Modified Eagle Medium (DMEM) (41966–029, Gibco) supplemented with 10% FBS (10270–106, Gibco) and 1% penicillin–streptomycin (P0781, Sigma-Aldrich), hereafter referred to as complete medium. All cell lines were cultured in standard incubators at 37 °C with 5% CO_2_, authenticated using short tandem repeat (STR) analysis and regularly tested for mycoplasma. To stably express lentiviral expression constructs, MDA-MB-231 and U87 cells were transduced with lentiviral supernatants at equal transducing units per ml in the presence of 8 μg/ml polybrene for 24 h. Transduced cells were selected with 4 μg/ml puromycin (ant-pr-1, InvivoGen) alone or combined with 2 mg/ml G418 disulfate salt solution (G8168, Sigma-Aldrich) for 3 or 4 days, respectively.

### Cell proliferation assay

A total of 3000 U87 or MDA-MB-231 cells were seeded in 96-well plates. After 24 h, medium was refreshed with complete medium only, or including DMSO (as a control) or NGI-1 (6652, R&D Systems) at the indicated concentrations. The well confluency was captured every 4 h at 10× magnification (5 images per well) for at least 5 days with the Incucyte S3 imaging system and analysed using the Incucyte S3 software (version 2020c). Logistic growth curve fitting was applied using GraphPad Prism software (version 9.5.0).

### Hypoxia and tunicamycin treatment

MDA-MB-231 (1.5 × 10^6^ cells/well) and U87 (1 × 10^6^ cells/well) cells were seeded in 6-well plates. After 24 h, medium was refreshed with complete medium only, or including DMSO (as a control) or tunicamycin (SML1287, Sigma-Aldrich) at the indicated concentrations. Before the cells were placed in the hypoxia chamber, cell confluency was captured at 10 × magnification (25 images per well) with the Incucyte S3 imaging system and analysed using the Incucyte S3 software (version 2020c). The cells were cultured at 37 °C in normoxia, or in hypoxia (1% O_2_, 5% CO_2_) in a Ruskinn InvivO_2_ 400 hypoxia workstation.

### Flow cytometry

MDA-MB-231 and U87 cells were trypsinised and collected at the indicated timepoints after transduction. Cells were stained in the dark with Concanavalin A, Alexa Fluor™ 647 Conjugate (1 μg/ml; C21421, Thermo Fisher Scientific) and Lens Culinaris (*Edible Lentil*) Agglutinin (LCA), fluorescein (FITC) (1:1,000; L32475, Thermo Fisher Scientific) for 1 h on ice in PBS supplemented with 2% FBS. The cells were thoroughly resuspended in the conjugated lectin mix. After staining, the cells were washed twice and at least 10,000 cells per sample were analysed using a NovoCyte 3000 Flow Cytometer (Acea Bioscience) with NovoExpress software version 1.4.1. Single cells were gated on size and shape using forward and side scatter. Data analysis was performed using FlowJo Software version 10.7.1.

### APEX2 proximity labelling and enrichment of biotinylated proteins

MDA-MB-231 cells stably expressing SP-APEX2 or the LOX-APEX2 variants (1 × 10^6^ cells/well) were embedded in 1.5 ml complete medium with 2.5% Matrigel (MG) growth factor reduced basement membrane matrix (354230, Corning) on top of a solidified layer of 50% MG in PBS in 12-well plates. Parental MDA-MB-231 cells were used as an additional control. After 24 h, APEX2 proximity labelling was performed as described previously^27^. Briefly, the top 2.5% MG layer was removed carefully and complete medium containing 500 μM biotinyl tyramide (biotin-phenol, SML2135, Sigma-Aldrich) was added for 30 min followed by the addition of 1 mM H_2_O_2_ (H1009, Sigma-Aldrich) for 60 s, promptly put on ice and washed three times with quencher solution. Cultrex Organoid Harvesting Solution (3700–100-01, R&D Systems) was used to harvest the cells according to the manufacturer’s protocol and the cell pellet was lysed with 2X cell lysis buffer (9803, Cell Signaling Technology), 2X Protease Inhibitor Cocktail (PI78429, Thermo Fisher Scientific) and the quencher solution as described in the protocol. The supernatants were quantified using the Pierce 660 nm protein assay kit (PI22660, Thermo Fisher Scientific) and a SpectraMax M5 plate reader. 100 µg protein was incubated with 20 µl Dynabeads™ MyOne™ Streptavidin C1 (65,001, Invitrogen) for 1 h at room temperature on a rotator, the beads were washed, and the biotinylated proteins eluted according to the protocol. The eluates were separated on a 4–12% Bis–Tris gradient gel (Invitrogen) for western blot or mass spectrometry (MS) analysis. Three independent biological replicates per sample were MS analysed.

### Gel preparation and in-gel digestion for MS analysis

The SDS-page gels were cut into 1 mm cubed pieces and washed for 3 × 20 min in 1 ml 200 mM ammonium bicarbonate and 40% (v/v) acetonitrile. The gel pieces were dehydrated and rehydrated three times in sequential 500 μl acetonitrile for 15 min followed by 500 μl of water for 15 min, followed by a final dehydration in acetonitrile. Gel pieces were rehydrated in 50 μl 9% (v/v) acetonitrile in 50 mM ammonium bicarbonate, and 20 ng/μl sequencing grade trypsin (Sigma-Aldrich) for 20 min, then a further 100 μl 9% (v/v) acetonitrile/50 mM ammonium bicarbonate was added, followed by incubation at 37° for 18 h. The samples were acidified by addition of 10 μl of 10% (v/v) formic acid and the isolated supernatant dried in a vacuum centrifuge at 40 °C for 30 min. The dried peptides were resuspended in 10 μl 0.1% formic acid (Sigma-Aldrich) in water for liquid chromatography coupled to tandem mass spectrometry (LC–MS/MS) analysis.

### Nano LC–MS/MS analysis

Peptides were analysed in an Ultimate 3000 RSLCnano system (Thermo Scientific) coupled to an Orbitrap Lumos mass spectrometer (Thermo Scientific). Peptides were injected onto a 50 cm long C18 Pepmap EasySpray column with an internal diameter of 75 μm and 2 μm particles (Thermo Scientific) at a flow rate of 300 nl per min. Peptides were separated at the same flow rate with a gradient of 1–27% Acetonitrile 0.1% formic acid over 30 min followed by 27–45% acetonitrile 0.1% formic acid over an additional 10 min. The emitter eluted the peptides directly into the MS source at a spray voltage of 1800 V. The mass spectrometer was operated in data dependent mode. An MS survey scan was acquired from m/z 350–1200 at a nominal resolution of 120,000 (defined at m/z 200) with a target value of 5e5 ions and a maximum fill time of 50 ms. The most intense multiply charged ions were selected for HCD MS2 analysis at a normalised collision energy of 29% followed by dynamic exclusion for 15 s with as many precursors selected per cycle as possible and a maximum time of 1.2 s.

### Proteomic data processing and data analysis

MS data analysis was performed with a combination of Progenesis LCMS (Nonlinear Dynamics) and Mascot (Matrix Science) with a 1% false discovery filter as a decoy database search. In brief, Progenesis LCMS generates extracted ion chromatograms of all detected features in the raw data and generates a mascot generic peak list file used for database searches. The extracted ion chromatograms facilitate further extraction of raw abundance values for all detected peptides. The mascot generic file peak list data was searched against a UniProt database of known human proteins. A protein is considered 'detected' only if two or more unique peptides derived from that protein are found in the MS data. Next, proteins with zero values across all samples and replicates were excluded, followed by computing average raw abundance values across the five technical replicates per biological replicate (3 in total) per sample. After which, the average abundance values were transformed using log_2_(x + 1) (to ensure 0 values remain zero after log_2_ transformation) and the log_2_ fold changes between samples were calculated (log_2_ FC = log_2_[A]−log_2_[B]). Finally, a *t* test was performed on the paired comparisons. *P* values were adjusted using the Benjamini-Hochberg (BH) method and the analysis was performed using R software version 3.5.1. Volcano plots were generated in GraphPad Prism (version 9.5.0) using the fold change of log_2_ transformed average abundance values per sample (compared to SP-APEX2 expressing cells) and −log_10_(raw *p* values) per protein. Proteins with log_2_ fold change > 1.5, and *p* value < 0.05 were included in further analysis.

### Nucleic acid isolation, PCR amplification and TIDE analysis

Genomic DNA was isolated using the DNeasy Blood & Tissue Kit (69504, Qiagen) according to the manufacturer’s protocol. PCR amplifications of *DDOST* exons 1 (sg*DDOST* #2) and 3 (sg*DDOST* #1) were performed with specific primers spanning the target sites and 100–200 ng genomic DNA template using Phusion® High-Fidelity DNA Polymerase (M0530, New England Biolabs) according to the manufacturer’s protocol. PCR reactions were purified by QIAquick PCR purification kit (28104, Qiagen) and subsequently subjected to Sanger Sequencing using the FW primers. CRISPR/Cas9-induced editing efficacy was quantified using the TIDE algorithm ^39^. Cells transduced with sgNon-targeting (sgNT) vectors were used as a negative control in all genomic DNA amplifications and TIDE outputs with at least R^2^ > 0.8 (preferably > 0.9) were considered. Quantification of indel frequencies were normalised to R^2^. Primers are listed in Supplementary Table 4.

### Protein isolation and immunoblotting

Cells were washed with ice cold PBS and protein lysates were made with 2X cell lysis buffer (9803, Cell Signaling Technology) and 2X Protease Inhibitor Cocktail (PI78429, Thermo Fisher Scientific) according to the manufacturer’s protocol. The supernatants were quantified using the Pierce 660 nm protein assay kit (PI22660, Thermo Fisher Scientific) and a SpectraMax M5 plate reader. Where indicated, the lysates were incubated with PNGase F (P0704, New England Biolabs) according to the manufacturer’s protocol. Equal amounts of proteins (typically 30 µg) were diluted in NuPage LDS sample buffer (NP0007, Thermo Fisher Scientific) with 2-mercaptoethanol (M6250, Sigma-Aldrich). The samples were incubated at 95 °C for 5 min, separated on a 4–12% Bis–Tris gradient gel (Invitrogen) and transferred onto a nitrocellulose membrane (1704159, Bio-Rad) using the Trans-Blot turbo transfer system (Bio-Rad) on Bio-Rad’s standardSD protocol settings. Membranes were blocked with intercept blocking buffer (927–70001, Li-COR) and incubated overnight at 4 °C with the primary antibodies in 2% intercept blocking buffer in PBS-T (0.05% Tween-20 in PBS). Membranes were washed and incubated with the secondary antibodies donkey anti-mouse IgG IRDye 680RD (1:5000; 926–68072, Li-COR), donkey anti-rabbit IgG IRDye 800CW (1:5000; 926–32213, Li-COR) in 2% intercept blocking buffer in PBS-T. Membranes were washed in PBS-T, captured using the Li-COR Odyssey CLx Infrared Imaging System and analysed using Image Studio Lite software version 5.2.5. Total LOX intensity bands were normalised to their internal vinculin control. For the fraction of LOX *N*-glycosylation, the intensity of fully *N*-glycosylated (FG) LOX (upper band) was divided by the total intensity of all LOX bands. The non-specific bands highlighted in the figures were excluded from quantification. Uncropped blots are included in Supplementary Information.

### Analysis of LOX-APEX2 in conditioned medium

Parental and MDA-MB-231 cells stably expressing SP-APEX2 or the LOX-APEX2 variants were seeded in 15 cm cell culture plates and grown to 80% confluency. The medium was removed, and 12 ml of fresh complete medium was added. After 24 h, the conditioned medium was collected on ice, protease and phosphatase inhibitors were added (1X final concentration, 78444, Thermo Fisher Scientific) and centrifuged at 1500 rpm for 5 min. V5 antibodies (1 µl/sample, R960-25, Thermo Fisher Scientific) were pre-bound to Protein G Sepharose ®, Fast Flow beads (30 µl/sample, P3296, Sigma) at 4 °C for 4 h on a rotator, then the beads were washed three times and added to the cleared conditioned medium in 15 ml centrifuge tubes and incubated overnight at 4 °C on a rotator. The beads were collected by centrifugation for 2 min at 2000 rpm, washed 3X and excess washing buffer was removed with a 25 gauge needle, and the beads were incubated in 30 µl 2X NuPage LDS sample buffer (NP0007, Thermo Fisher Scientific) with 2-mercaptoethanol (M6250, Sigma-Aldrich) at 95 °C for 10 min. The samples were separated on a 4–12% Bis–Tris gradient gel (Invitrogen), followed by standard immunoblotting. Membranes were incubated with the V5 antibody (1:1000; R960-25, Thermo Fisher Scientific) and detected using Alexa Fluor® 790 AffiniPure Goat Anti-Mouse IgG, light chain specific (1:20,000; 115–655-174, Jackson ImmunoResearch).

### KEGG pathway analysis

The Uniprot protein names were matched with their official gene symbol. Entrez IDs were retrieved using org.Hs.eg.db (3.15.0) and mapped using AnnotationDbi (1.58.0). KEGG pathway analysis was performed with EnrichR (3.1) and the “KEGG_2021_Human” database^63^. Fold changes were visualised with Pathview (1.36.1). Analysis was performed by R (4.2.0) in RStudio Workbench (2022.02.3 build 492.pro3).

### Antibodies

The following antibidies were used: Cas9 (ab191468, Abcam), GAPDH (1:10,000; CB1001, Millipore), GFP (ab6556, Abcam), LOX (L4794, Sigma-Aldrich), OST48 (sc-74408, Santa Cruz Biotechnology), RPN1 (sc-48367, Santa Cruz Biotechnology), RPN2 (sc-166421, Santa Cruz Biotechnology), streptavidin IRDye 800CW (926–32230, Li-COR), vinculin (4650, Cell Signaling Technology) and V5 (R960-25, Thermo Fisher Scientific). All antibodies were diluted 1:1000 in 2% intercept blocking buffer in PBS-T (0.05%), unless stated otherwise.

#### URLs

AlphaFold, https://alphafold.ebi.ac.uk/.

ClinVar, https://www.ncbi.nlm.nih.gov/clinvar/.

STRING version 11.5, https://string-db.org/.

TIDE, http://tide.nki.nl.

### Statistical analysis

Graphs and error bars represent mean ± SEM, unless stated otherwise. All statistical tests were performed using GraphPad Prism software (version 9.5.0). *P* < 0.05 was considered statistically significant. Except for *P* < 0.001 and *P* ≥ 0.05, exact *P* values are always shown in the figures, corresponding figure panels or, where indicated, in Supplementary Table 1. *P* values are determined by unpaired two-tailed Student’s *t* tests, unless stated otherwise. The adjusted *P* values (q values) were determined by unpaired two-tailed Student’s *t* test with FDR multiple testing correction using the two-stage step-up method (Benjamini, Krieger, and Yekutieli) in GraphPad Prism software. For the proliferation curves, we performed logistic growth curve fitting constraining the maximum population/confluency (YM) to 100% comparing independent fits of three curves (allowing different rate constants [k] for each curve) with a global fit that shares k, using a (extra sum-of-squares) F test in GraphPad Prism software. We repeated this approach with pair wise comparisons of the three curves.

### Supplementary Information

Supplementary Information 1.

Supplementary Information 2.

Supplementary Table 1.

Supplementary Table 2.

Supplementary Table 3.

Supplementary Table 4.

## Data Availability

All study data are included in the article and/or Supplementary Information files. The mass spectrometry proteomics data have been deposited to the ProteomeXchange Consortium via the PRIDE partner repository with the dataset identifier PXD042959 and https://doi.org/10.6019/PXD042959.
