# Supplementary material for: Functional classification of DDOST variants of uncertain clinical significance in congenital disorders of glycosylation
Source: Sci Rep. 2023 Oct 17;13:17648. doi: 10.1038/s41598-023-42178-y (PMC10582084; doi:10.1038/s41598-023-42178-y)

Functional Classification of *DDOST* Variants of Uncertain Clinical Significance in Congenital Disorders of Glycosylation

Sjors M. Kas, Piyushkumar A. Mundra, Duncan L. Smith, Richard Marais

Supplementary Information (SI)

The original images of the western blots used in this study.

This document contains all the uncropped western blots that are presented in the manuscript. The membranes were captured using the Li-COR Odyssey CLx Infrared Imaging System using a 700 nm (shown in red) and 800 nm (green) channel, which enabled the detection of proteins with similar molecular weight on a single membrane. The white rectangles mark the cropped images that are shown in the manuscript. A few membranes were reprobed with new antibodies after the first round of incubations.

Fig. 2c

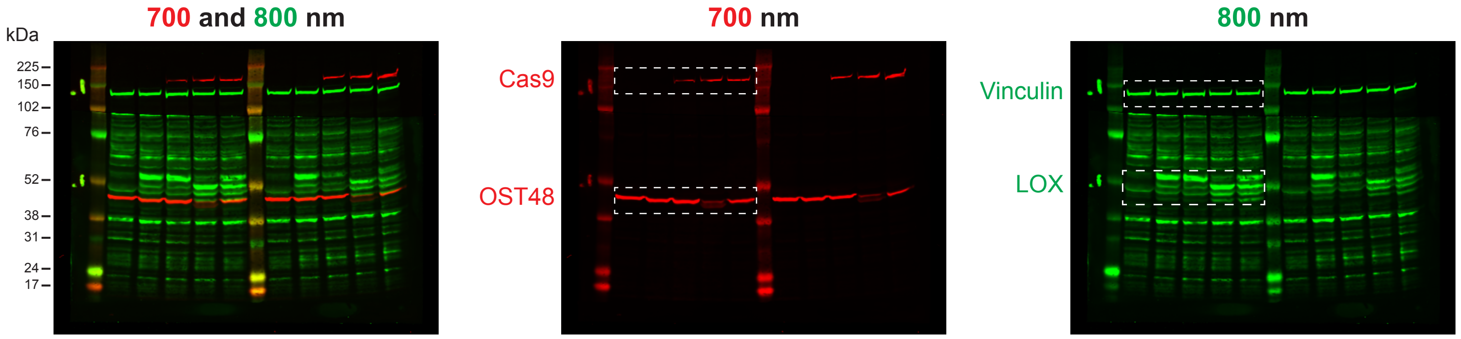

Fig. 2d

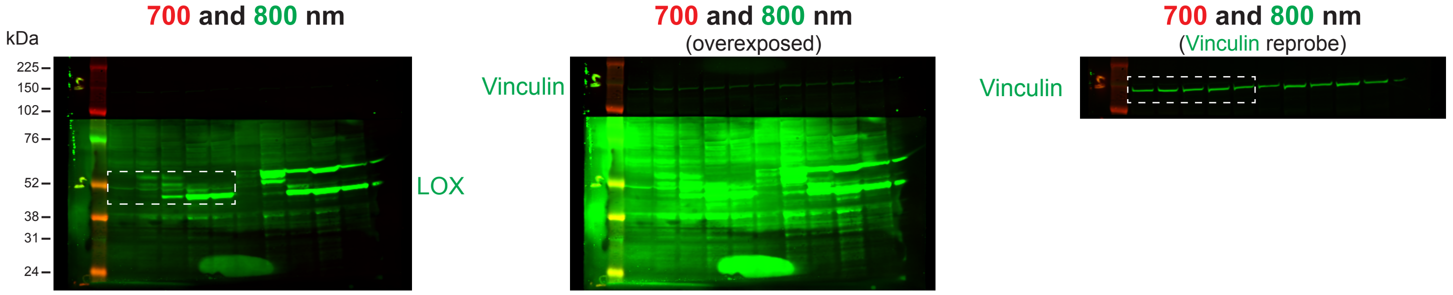

Fig. 2e

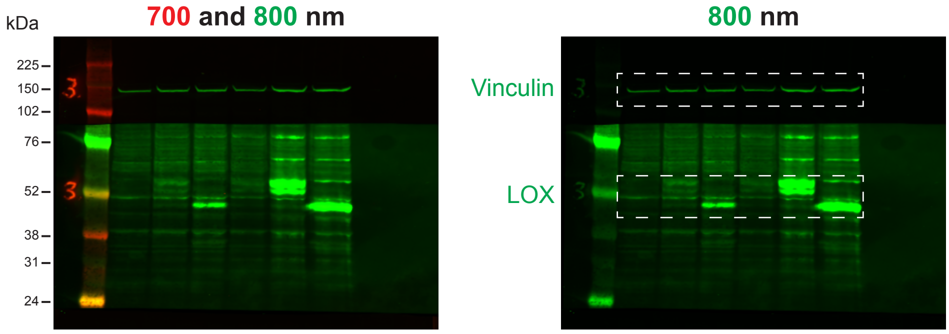

Fig. 3b

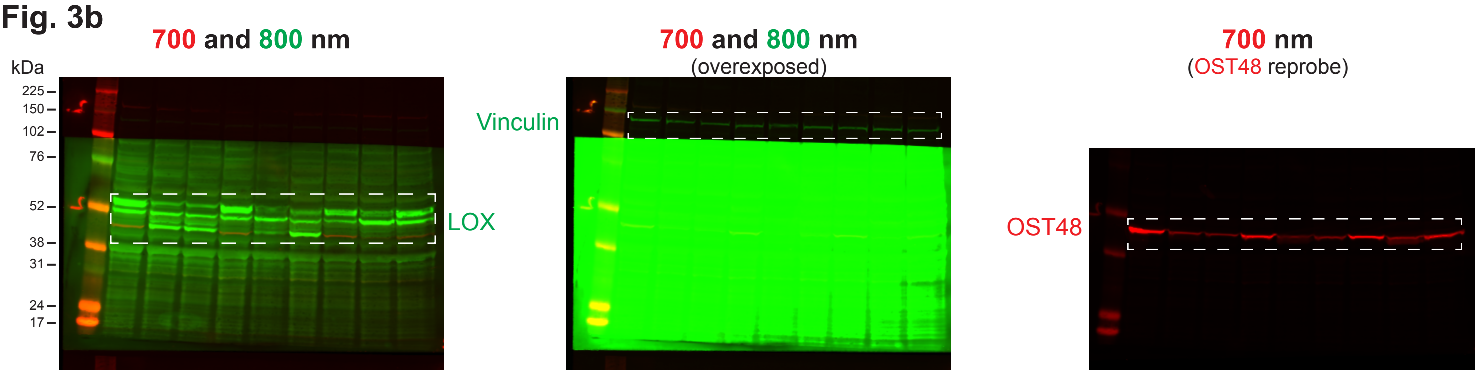

Fig. 4a

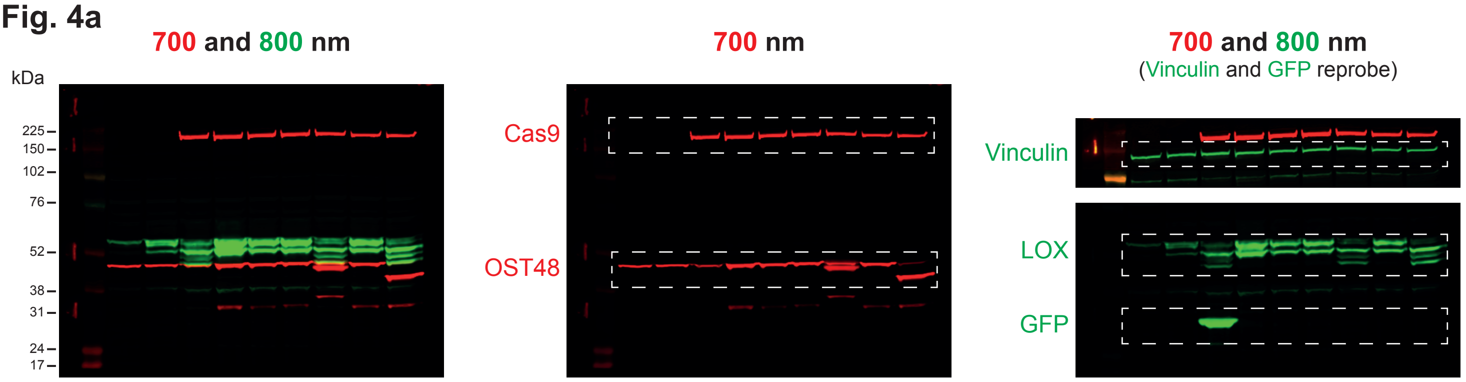

Fig. 4e

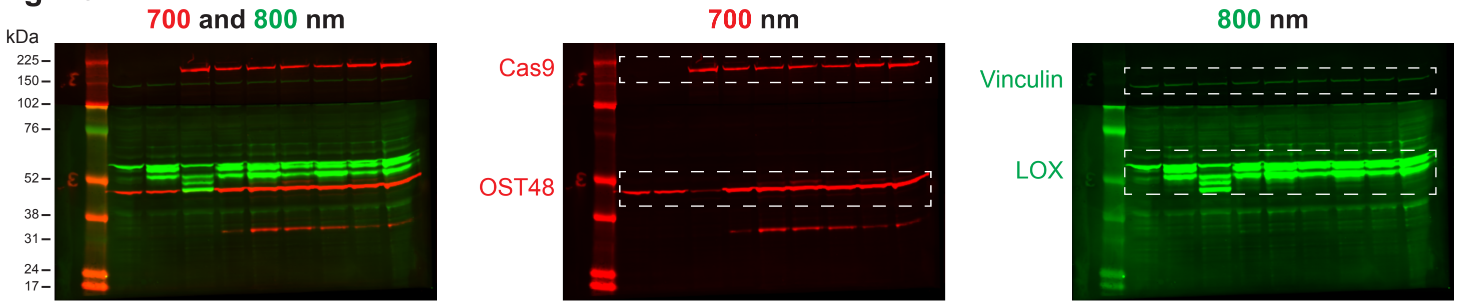

Fig. 4f

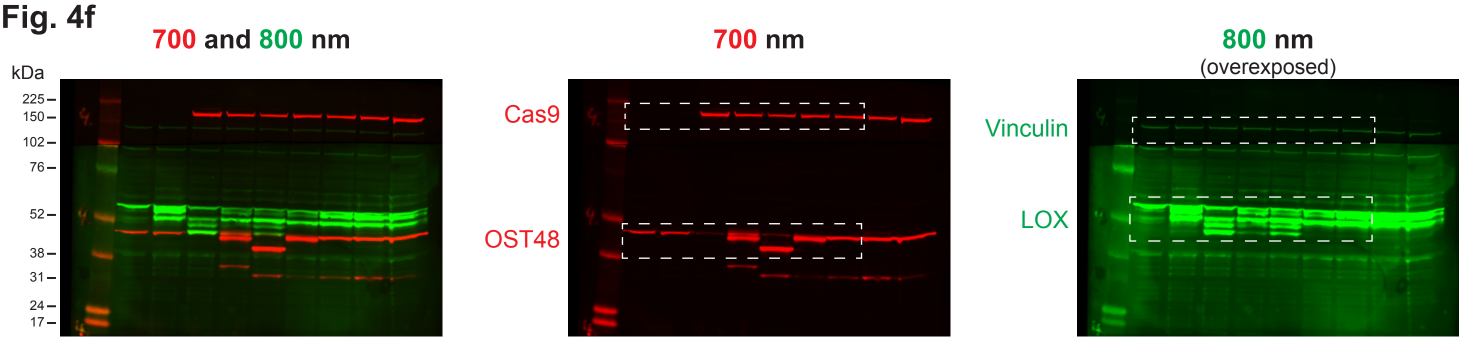

Fig. 5: Top left

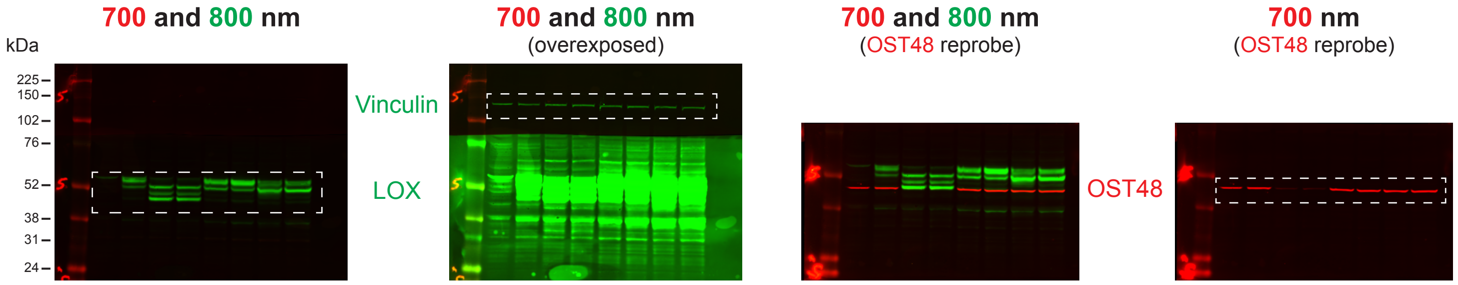

Fig. 5: Top right

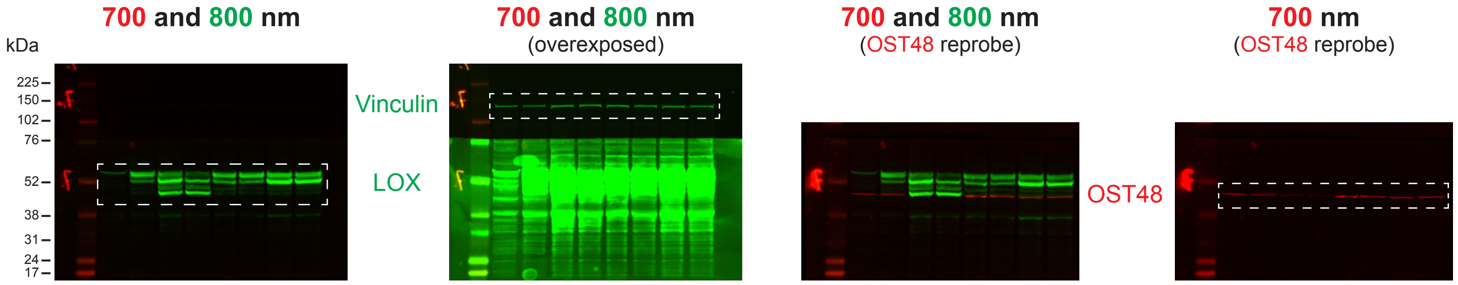

Fig. 5: Bottom left

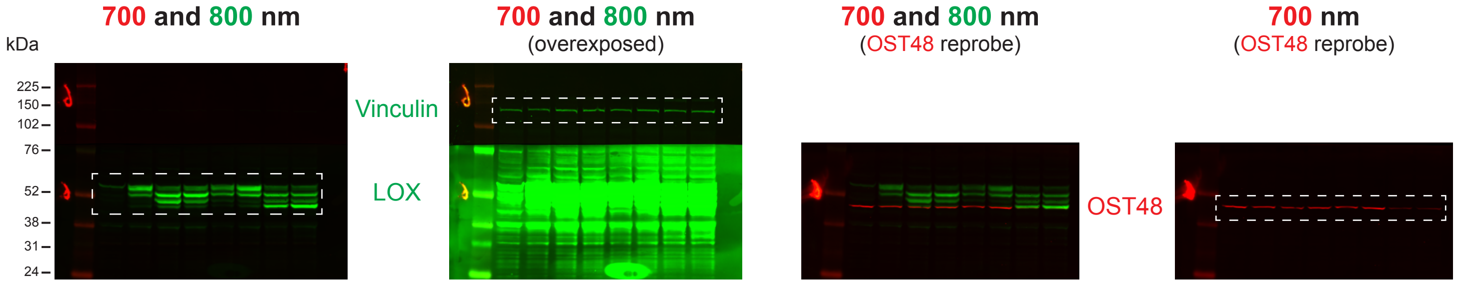

Fig. 5: Bottom right

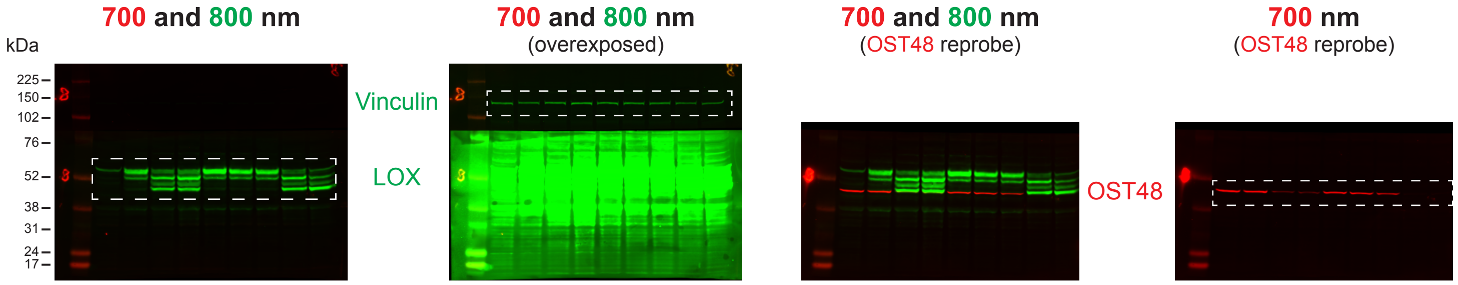

Supplementary Fig. 2a

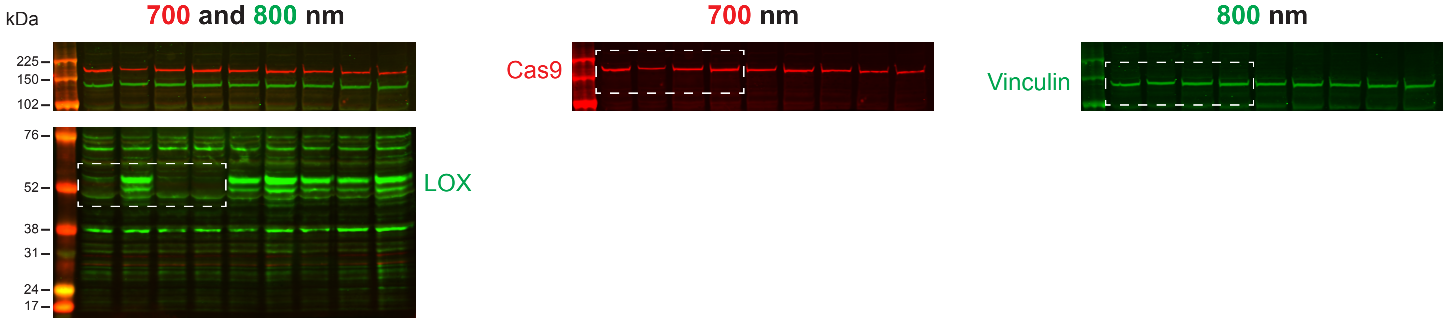

Supplementary Fig. 2b

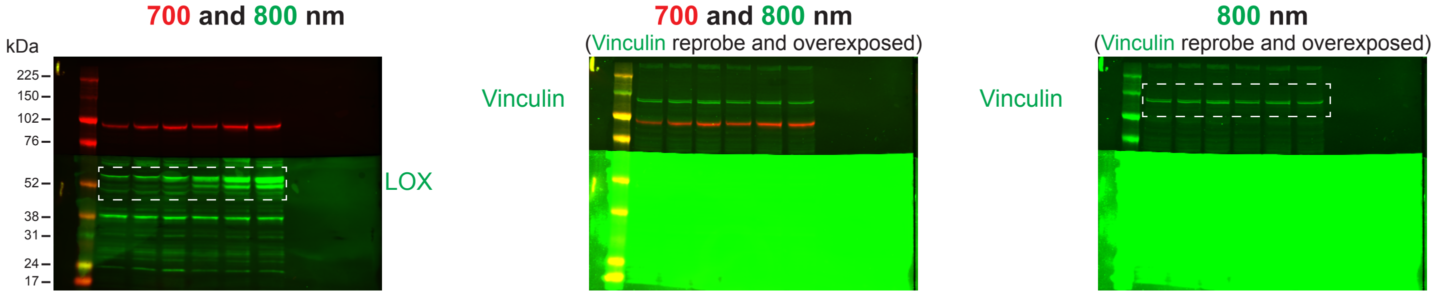

Supplementary Fig. 2c

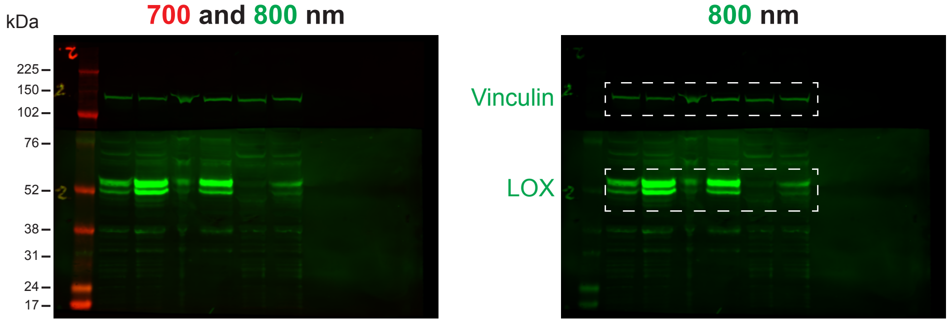

Supplementary Fig. 2d

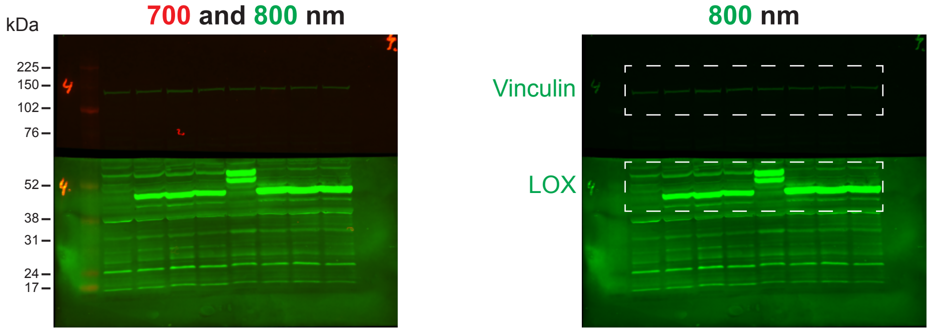

Supplementary Fig. 3b: MDA-MB-231

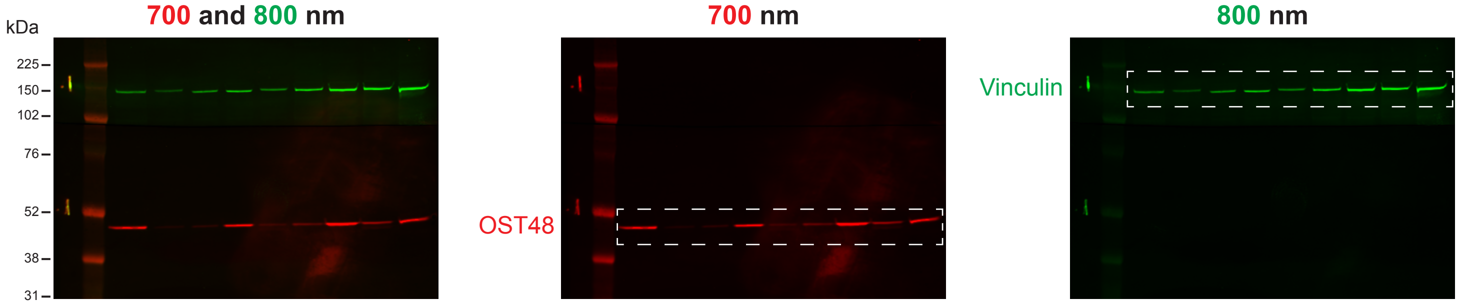

Supplementary Fig. 3b: U87

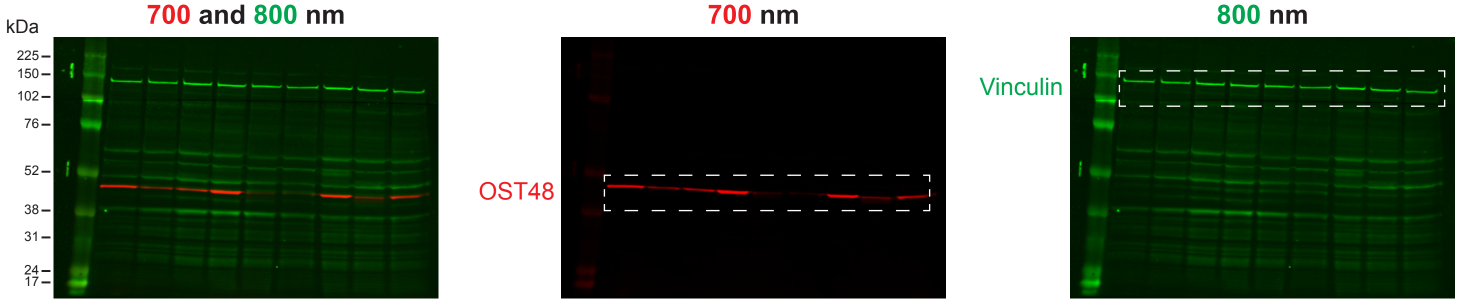

Supplementary Fig. 4a

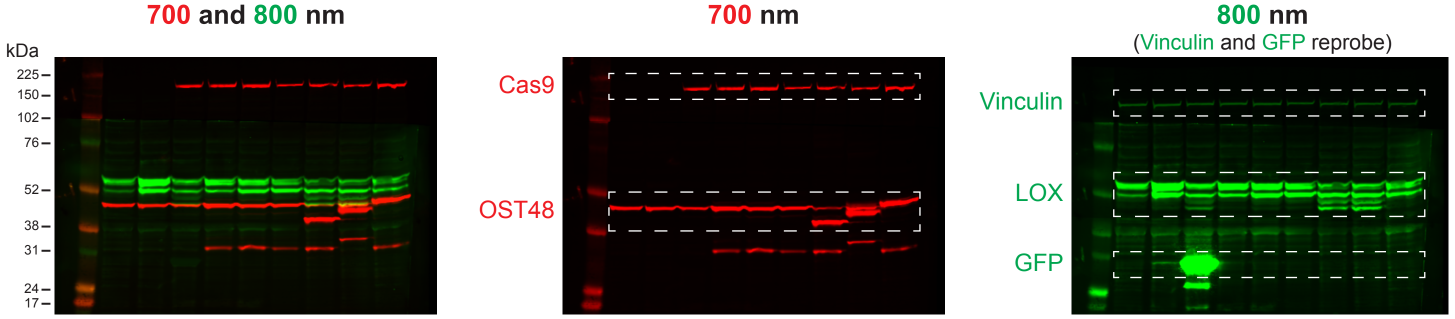

Supplementary Fig. 5a: Top left

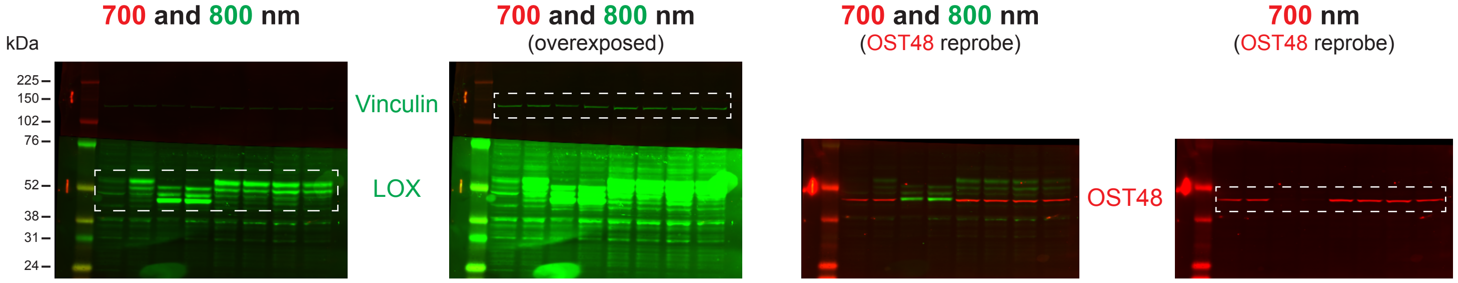

Supplementary Fig. 5a: Top right

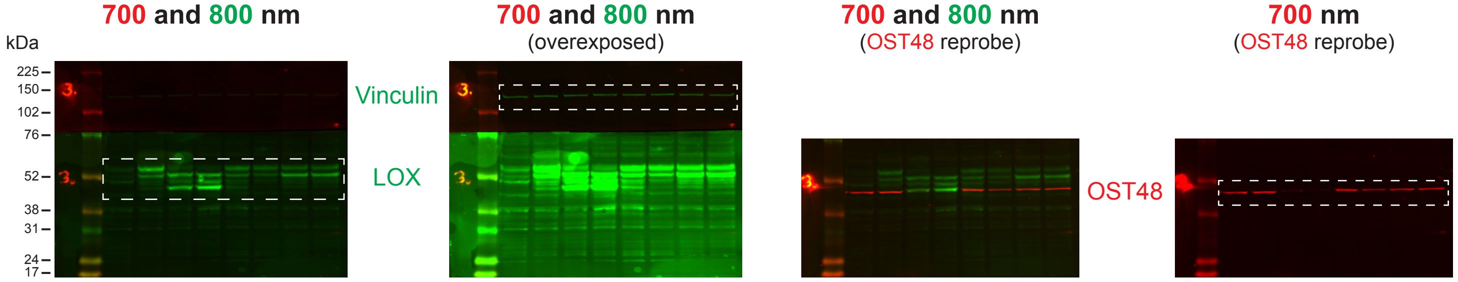

Supplementary Fig. 5a: Bottom left

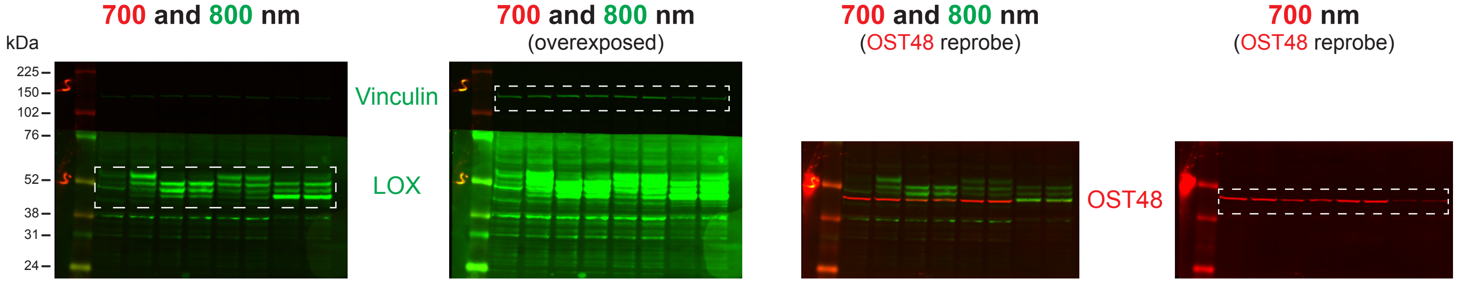

Supplementary Fig. 5a: Bottom right

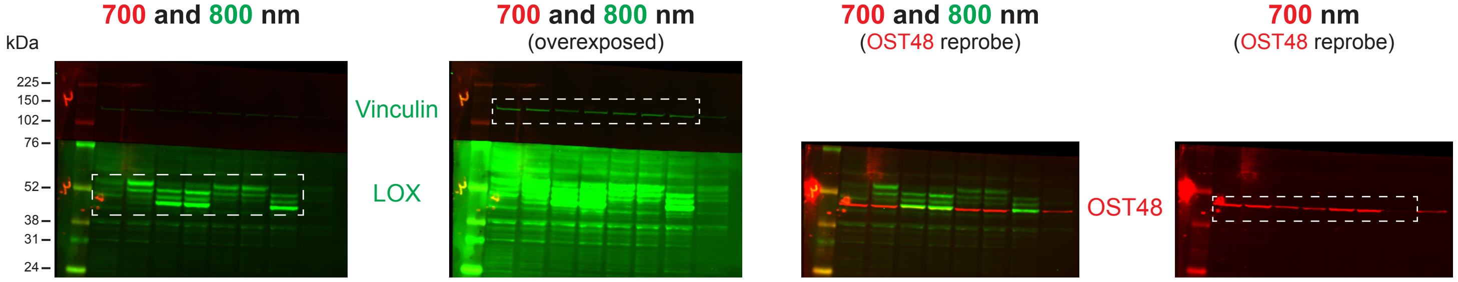

Supplementary Fig. 5b

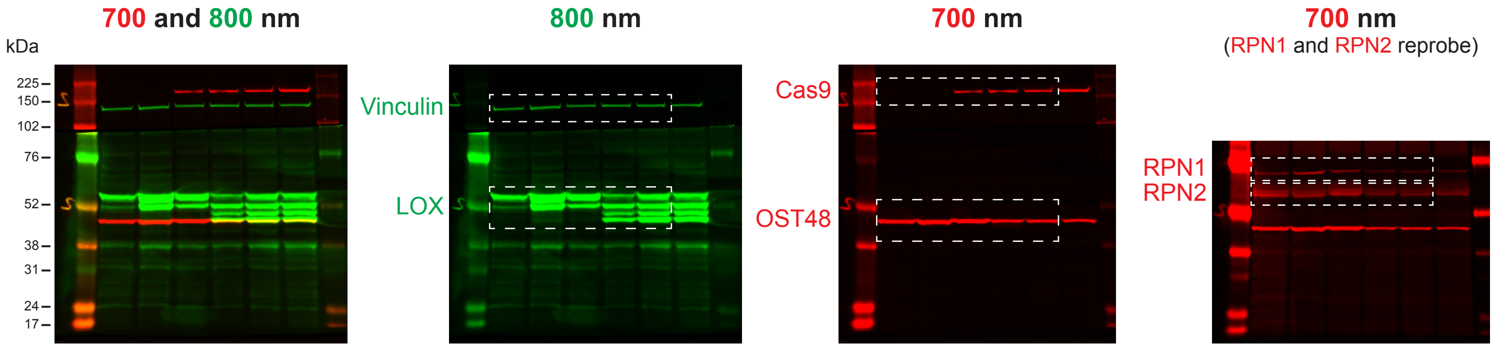

Supplementary Fig. 5c

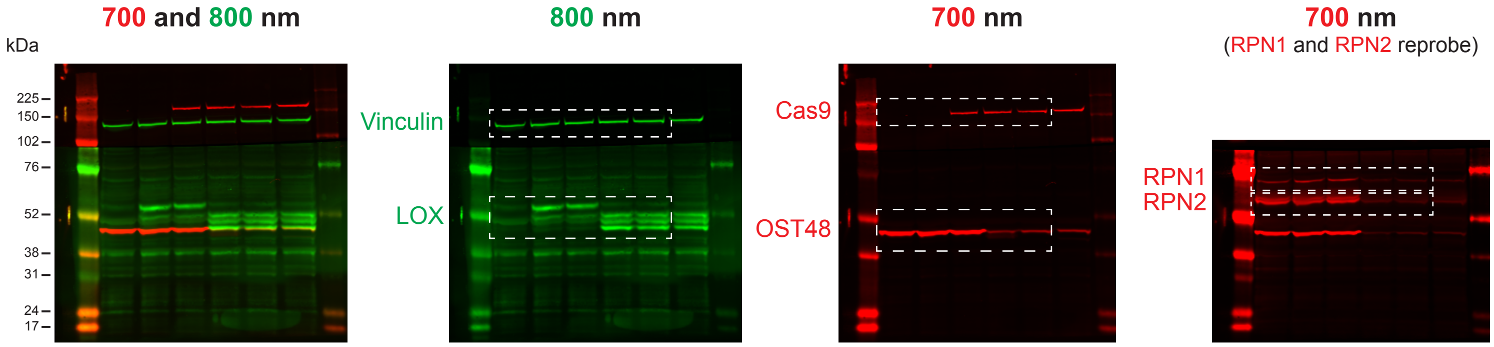

Supplement: Supplementary file 1 — Supplementary Information 1. [file 41598_2023_42178_MOESM1_ESM.pdf]
