# Supplementary material for: Functional classification of DDOST variants of uncertain clinical significance in congenital disorders of glycosylation
Source: Sci Rep. 2023 Oct 17;13:17648. doi: 10.1038/s41598-023-42178-y (PMC10582084; doi:10.1038/s41598-023-42178-y)
Supplement: Supplementary file 2 — Supplementary Information 2. [file 41598_2023_42178_MOESM2_ESM.pdf]

Functional Classification of *DDOST* Variants of Uncertain Clinical Significance in Congenital Disorders of Glycosylation

Sjors M. Kas, Piyushkumar A. Mundra, Duncan L. Smith, Richard Marais

Supplementary Figures

Supplementary Fig. 1. LOX interacting proteins are enriched for protein processing in the endoplasmic reticulum.

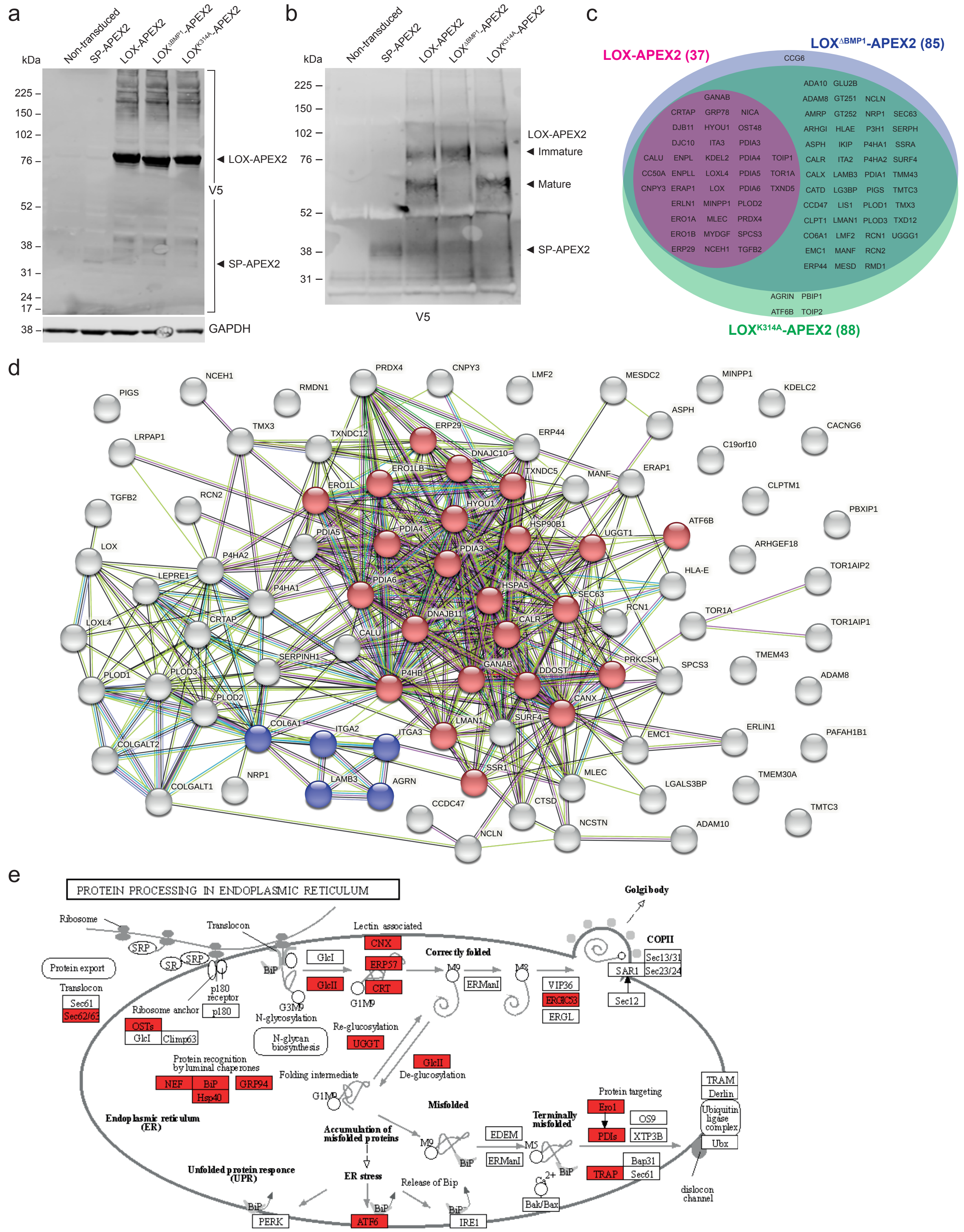

(a) Western blot for the V5 epitope tag in MDA-MB-231 cells stably expressing SP-APEX2, LOX-APEX2, LOX$^{\Delta BMP1}$-APEX2 and LOX^{K314A}-APEX2. GAPDH: loading control.

(b) Western blot for V5-tagged proteins captured by V5 immunoprecipitation of conditioned medium from MDA-MB-231 cells stably expressing SP-APEX2, LOX-APEX2, LOX$^{\Delta BMP1}$-APEX2 and LOX^{K314A}-APEX2.

(c) Venn diagram showing the overlap of all significant LOX-interactors (89 proteins) between MDA-MB-231 cells stably expressing LOX-APEX2 (37; magenta), LOX$^{\Delta BMP1}$-APEX2 (85; blue) and LOX^{K314A}-APEX2 (88; green).

(d) Projection of all significant LOX-interactors (89 proteins) onto the STRING protein–protein interaction network (version 11.5). LOX-interactors involved in “protein processing in endoplasmic reticulum” or “ECM-receptor interaction” according to KEGG pathway enrichment analysis are highlighted in red and blue respectively.

(e) Projection of LOX interacting proteins involved in protein processing in the endoplasmic reticulum (hsa04141) are highlighted in Pathview (red) according to KEGG pathway enrichment analysis[1].

Supplementary Fig. 2. Hypoxia induced LOX expression is cell type and density dependent, and cell surface *N*-glycan levels are reduced after *DDOST* depletion.

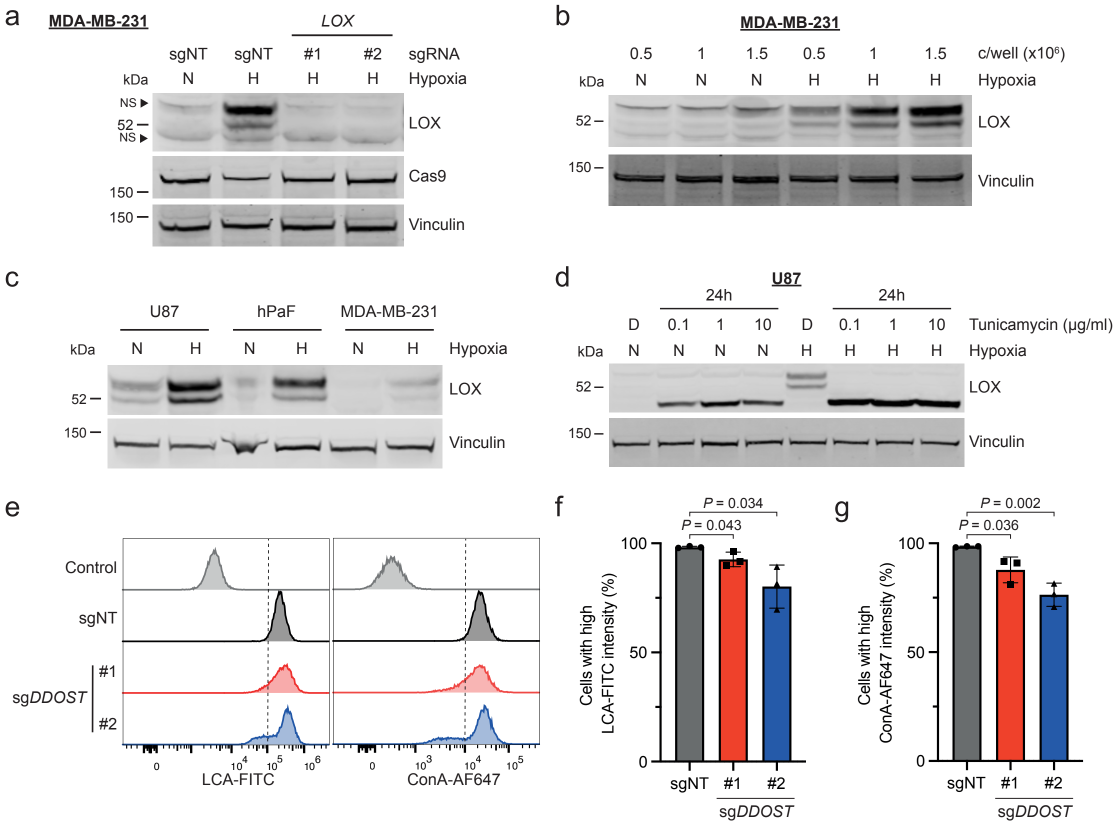

(a) Western blot for LOX, Cas9 and vinculin (loading control) in MDA-MB-231 transduced with two independent LentiCRISPRv2-sg*LOX* vectors (#1, #2) and a sgNT control. Cells were re-seeded 10 days after gene targeting, then moved to normoxia (N) or hypoxia (H; 1% O_2) on day 11 for 24 hr. Image is representative of 3 independent experiments. Arrowheads highlight two non-specific (NS) bands.

(b) Western blot for LOX and vinculin (loading control) in MDA-MB-231 cells seeded at different densities and cultured in normoxia (N) or hypoxia (H; 1% O_2) for 24 hr. Image is representative of 3 independent experiments.

(c) Western blot for LOX and vinculin (loading control) in human primary pancreatic fibroblasts (hPaF), U87 and MDA-MB-231 cells cultured in normoxia (N) or hypoxia (H; 1% O_2) for 24 hr. Image is representative of 3 independent experiments.

(d) Western blot for LOX and vinculin (loading control) in U87 cells treated with the indicated concentrations of tunicamycin or DMSO (D) control and incubated in normoxia (N) or hypoxia (H; 1% O_2) for 24 hr. Image is representative of 3 independent experiments.

(e) Histograms of LCA and ConA binding in MDA-MB-231 cells 14 days after CRISPR/Cas9-mediated gene editing with sg*DDOST* (#1, #2) or sgNT. The control shows unstained cells and data are representative of 3 independent experiments.

(f, g) Quantification of LCA (f) and ConA (g) binding in MDA-MB-231 cells 14 days after CRISPR/Cas9-mediated gene editing with sg*DDOST* (#1, #2) or sgNT. Data are mean ± s.d. of 3 independent experiments.

Supplementary Fig. 3. OST48 is essential for MDA-MB-231 cell proliferation.

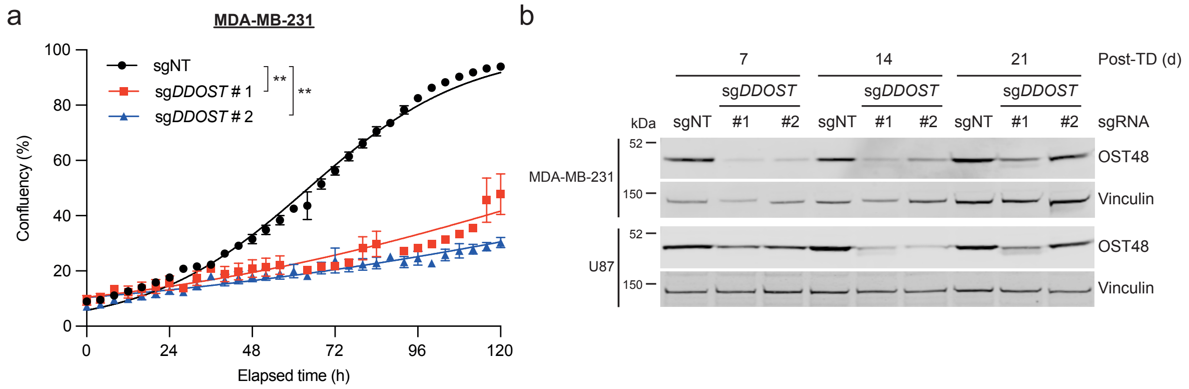

(a) Proliferation of MDA-MB-231 cells after CRISPR/Cas9-mediated gene editing with sg*DDOST* (#1, #2) or sgNT. Cells were re-seeded 7 days after transduction and proliferation was determined from day 8 for 120 hours using real-time IncuCyte imaging. Data are mean ± SEM of 3 independent experiments with three technical repeats per condition. ** P <0.001; F test for comparison of logistic growth curve fits.
(b) Western blot for OST48 and vinculin (loading control) in MDA-MB-231 and U87 cells 7, 14 and 21 days (d) after CRISPR/Cas9-mediated gene editing with sg*DDOST* (#1, #2) or sgNT. Data are representative of 3 independent experiments.

Supplementary Fig. 4. Identification of clinically relevant *DDOST* variants.

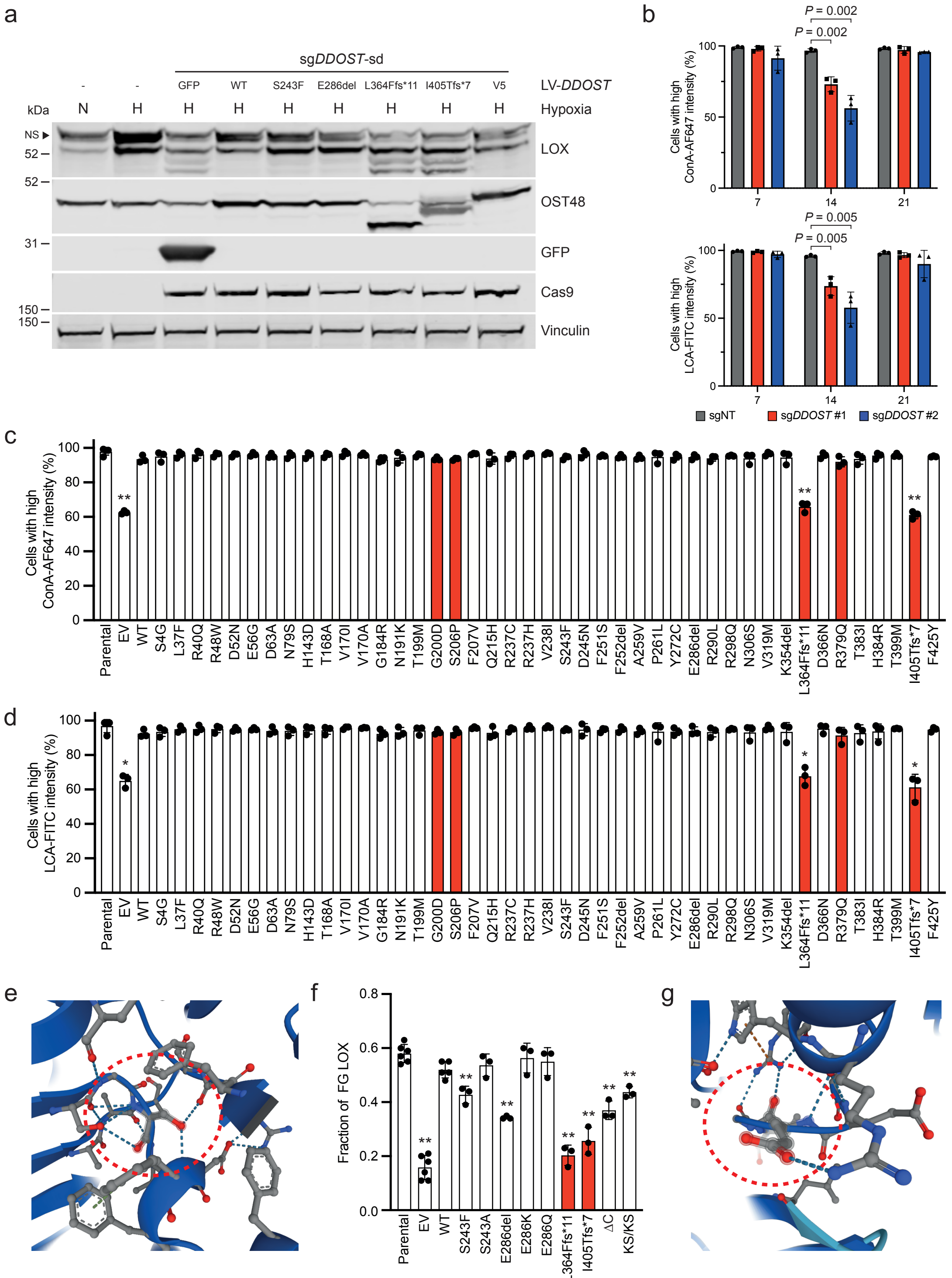

(a) Western blot for LOX, OST48, GFP, Cas9 and vinculin (loading control) in U87 cells after CRISPR/Cas9 gene editing with sg*DDOST*-sd and reconstitution with GFP, *DDOST* (WT) and the indicated *DDOST* cDNA variants. Cells were re-seeded 7 days after transduction, then transferred to normoxia (N) or hypoxia (H; 1% O_2) on day 8 for 24 hr. Data are representative of 3 independent experiments. Arrowhead highlights non-specific (NS) band.

(b) Quantification of ConA (top) and LCA (bottom) binding in U87 cells 7, 14 and 21 days after CRISPR/Cas9 gene editing with sg*DDOST* (#1, #2) or sgNT. Data are mean ± s.d. of 3 independent experiments. ** *P* values <0.001.

(c, d) Quantification of ConA (c) and LCA (d) binding in U87 cells after CRISPR/Cas9 gene editing with sg*DDOST*-sd and reconstitution with empty vector (EV, control), *DDOST* (WT) and the 43 *DDOST* cDNA variants indicated. Parental cells are shown for comparison. Cells were analysed 14 days after transduction. Variants in red were reported previously[2–4]. Data are mean ± s.d. of 3 independent transductions, compared to WT: * adjusted *P* values <0.05 (*) and <0.01 (**); unpaired two-tailed Student's *t* tests with FDR multiple testing correction.

(e) Image depicting AlphaFold structure prediction for OST48 (A0A0C4DGS1) in the region of S243 (red dotted circle). Blue dotted lines: hydrogen bonds. Carbon, nitrogen and oxygen atoms are coloured in grey, blue or red, respectively.

(f) Quantification of fully *N*-glycosylated (FG) LOX as a fraction of total LOX in *DDOST*-depleted U87 cells reconstituted with EV, *DDOST* (WT) or the *DDOST* mutants indicated. Parental cells are shown for comparison. Cells were re-seeded 7 days after transduction and transferred to hypoxia (1% O_2) on day 8 for 24 hr. Variants in red were reported previously[2–4]. Variants were compared to WT: ** adjusted *P* values <0.001; unpaired two-tailed Student's *t* tests with FDR multiple testing correction. Data are mean ± s.d. of 3 independent experiments.

(g) Image depicting AlphaFold structure prediction for OST48 (A0A0C4DGS1) in the region of E286 (red dotted circle). Blue dotted lines: hydrogen bonds. Carbon, nitrogen and oxygen atoms are coloured in grey, blue or red, respectively.

Supplementary Fig. 5. OST complex stability depends on all common OST subunits.

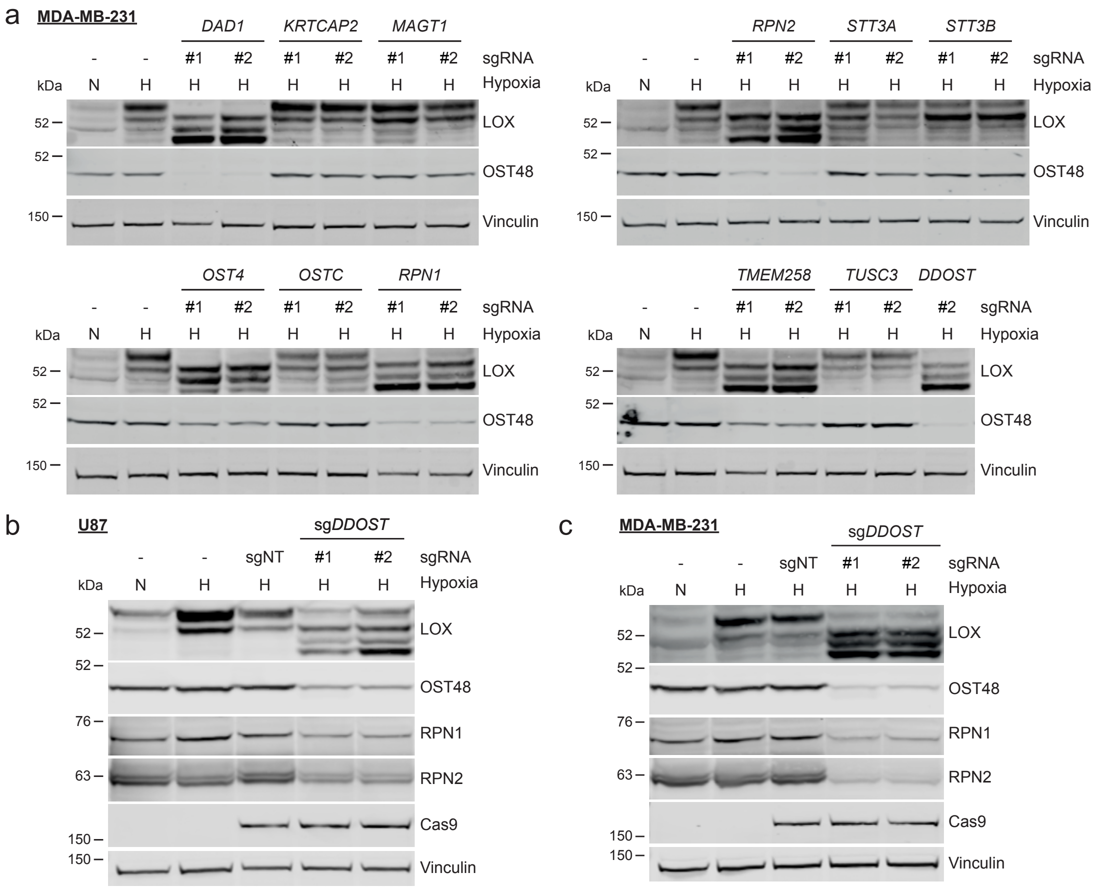

(a) Immunoblot for LOX, OST48 and vinculin (loading control) in MDA-MB-231 cells 12 days after CRISPR/Cas9-mediated targeting of the indicated OST genes. Cells were re-seeded 10 days after transduction, then moved to normoxia (N) or hypoxia (H; 1% O_2) on day 11 for 24 hr.

(b, c) Immunoblots for LOX, OST48, RPN1, RPN2, Cas9 and vinculin (loading control) in U87 (b) and MDA-MB-231 (c) cells 9 days after CRISPR/Cas9 gene editing with sg*DDOST* (#1, #2) or sgNT. Cells were re-seeded 7 days after transduction, then moved to normoxia (N) or hypoxia (H; 1% O_2) on day 8 for 24 hr.

References

1. Kanehisa, M., Furumichi, M., Sato, Y., Kawashima, M. & Ishiguro-Watanabe, M. KEGG for taxonomy-based analysis of pathways and genomes. *Nucleic Acids Res.* **51**, D587–D592 (2023).
2. Jones, M. A. *et al.* DDOST Mutations Identified by Whole-Exome Sequencing Are Implicated in Congenital Disorders of Glycosylation. *Am. J. Hum. Genet.* **90**, 363–368 (2012).
3. Pi, S. *et al.* The second DDOST-CDG patient with lactose intolerance, developmental delay, and situs inversus totalis. *J. Hum. Genet.* **67**, 103–106 (2022).
4. Elsharkawi, I. *et al.* DDOST-CDG : Clinical and Molecular Characterization of a Third Patient with a Milder and a Predominantly Movement Disorder Phenotype. *J. Inherit. Metab. Dis.* jimd.12565 (2022) doi:10.1002/jimd.12565.
